# Body-mass index, blood pressure, and cause-specific mortality in India: a prospective cohort study of 500 810 adults

**DOI:** 10.1016/S2214-109X(18)30267-5

**Published:** 2018-06-13

**Authors:** Vendhan Gajalakshmi, Ben Lacey, Vendhan Kanimozhi, Paul Sherliker, Richard Peto, Sarah Lewington

**Affiliations:** a Epidemiological Research Centre, Chennai, Tamil Nadu, India; b MRC Population Health Research Unit, Nuffield Department of Population Health, University of Oxford, Oxford, UK; c Clinical Trial Service Unit and Epidemiological Studies Unit, Nuffield Department of Population Health, University of Oxford, Oxford, UK; d NIHR Oxford Biomedical Research Centre, Oxford, UK

## Abstract

**Background:**

The association between cause-specific mortality and body-mass index (BMI) has been studied mainly in high-income countries. We investigated the relations between BMI, systolic blood pressure, and mortality in India.

**Methods:**

Men and women aged 35 years or older were recruited into a prospective study from the general population in Chennai, India between Jan 1, 1998, and Dec 31, 2001. Participants were interviewed (data collected included age, sex, education, socioeconomic status, medical history, tobacco smoking, and alcohol intake) and measured (height, weight, and blood pressure). Deaths were identified by linkage to Chennai city mortality records and through active surveillance by household visits from trained graduate non-medical fieldworkers. After the baseline survey, households were visited once in 2002–05, then biennially until 2015. During these repeat visits, structured narratives of any deaths that took place before March 31, 2015, were recorded for physician coding. During 2013–14, a random sample of participants was also resurveyed as per baseline to assess long-term variability in systolic blood pressure and BMI. Cox regression (standardised for tobacco, alcohol, and social factors) was used to relate mortality rate ratios (RRs) at ages 35–69 years to systolic blood pressure, BMI, or BMI adjusted for usual systolic blood pressure.

**Findings:**

500 810 participants were recruited. After exclusion of those with chronic disease or incomplete data, 414 746 participants aged 35–69 years (mean 46 [SD 9]; 45% women) remained. At recruitment, mean systolic blood pressure was 127 mm Hg (SD 15), and mean BMI was 23·2 kg/m^2^ (SD 3·8). Correlations of resurvey and baseline measurements were 0·50 for systolic blood pressure and 0·88 for BMI. Low BMI was strongly associated with poverty, tobacco, and alcohol. Of the 29 519 deaths at ages 35–69 years, the cause was vascular for 14 935 deaths (12 504 cardiac, 1881 stroke, and 550 other). Vascular mortality was strongly associated with systolic blood pressure: RRs per 20 mm Hg increase in usual systolic blood pressure were 2·45 (95% CI 2·16–2·78) for stroke mortality, 1·74 (1·64–1·84) for cardiac mortality, and 1·84 (1·75–1·94) for all vascular mortality. Although BMI strongly affected systolic blood pressure (an increase of about 1 mm Hg per kg/m^2^) and diabetes prevalence, BMI was little related to cardiac or stroke mortality, with only small excesses even for grade 1 obesity (ie, BMIs of 30·0–35·0 kg/m^2^). After additional adjustment for usual systolic blood pressure, BMI was inversely related to cardiac and stroke mortality throughout the range 15·0–30·0 kg/m^2^: when underweight participants (ie, BMI 15·0–18·5 kg/m^2^) were compared with overweight participants (ie, BMI 25·0–30·0 kg/m^2^), the blood-pressure-adjusted RR was 1·28 (95% CI 1·20–1·38) for cardiac mortality and 1·46 (1·22–1·73) for stroke mortality.

**Interpretation:**

In this South Asian population, BMI was little associated with vascular mortality, even though increased BMI is associated with increased systolic blood pressure, which in turn is associated with increased vascular mortality. Hence, some close correlates of below-average BMI must have important adverse effects, which could be of relevance in all populations.

**Funding:**

UK Medical Research Council, British Heart Foundation, Cancer Research UK.

## Introduction

A key target adopted by the WHO World Health Assembly in 2013^1^ to help to limit mortality from non-communicable diseases was halting the worldwide increase in overweight and obesity (defined by WHO as body-mass index [BMI] of 25–30 kg/m^2^ and ≥30 kg/m^2^, respectively). The 2015 UN Sustainable Development Goals^2^ subsequently included prevention of non-communicable diseases as a global goal.

The relevance of BMI to non-communicable disease mortality has, however, not been well characterised in low-income or lower-middle-income countries, where half the world's population lives. In 2015, estimates of the burden of BMI-related mortality in such populations^3^ were based on the Global BMI Mortality Collaboration meta-analyses of 239 prospective studies.^4^ But most of those studies were of European-origin populations. None was African, and only three^5^, ^6^, ^7^ were South Asian. On average, the risk of death was lowest in the BMI range 20–25 kg/m^2^ and was increased by half both in the 15–18·5 kg/m^2^ (which WHO classifies as underweight) and 30–35 kg/m^2^ (classified as grade 1 obesity) ranges.

**Research in context**

**Evidence before this study**

A 2015 literature search by the Global BMI Mortality Collaboration identified 239 prospective studies of body-mass index (BMI). Only three were South Asian (in Mumbai, rural Kerala, and Bangladesh, respectively). Although the South Asian studies suggested little association between BMI and vascular mortality, they included too few participants for statistical stability. By contrast, in the other 236 studies, which were mostly done in Western populations and included 10 million participants, cardiac mortality was strongly related to BMI: it was minimal at 20–22·5 kg/m^2^ and approximately doubled with each increase of 10 kg/m^2^. Four large studies of BMI, blood pressure, lifestyle factors, and mortality began in the 1990s in India—the studies in Mumbai and rural Kerala mentioned previously, one in Trivandrum that has yet to report results, and this study in Chennai.

**Added value of this study**

Ours is the largest Indian study reported so far of BMI and mortality. It more than triples the amount of prospective evidence on BMI and adult mortality in South Asians, producing statistically stable results for all BMI categories from underweight (15·0–18·5 kg/m^2^) to grade 1 obesity (30·0–35·0 kg/m^2^), although not for more severe obesity. Our findings show that blood pressure is a strong determinant of vascular mortality and that BMI is a strong determinant of blood pressure. But despite these associations, throughout the wide BMI range 15·0–35·0 kg/m^2^ there is little association between BMI and cardiac or other vascular mortality.

**Implications of all the available evidence**

Our findings suggest that the important adverse effects of BMI on blood pressure are counterbalanced by important adverse effects on vascular mortality of some correlates of lower BMI, which, if understood, might be of relevance in all populations. If, however, these correlates are mainly poverty-related developmental factors that act before adulthood to affect BMI and vascular risk, then the adverse effects of becoming overweight in adult life could be about as great in South Asia as elsewhere.

The mechanisms underlying the excess mortality associated with underweight are not well understood. In European-origin populations, the excess mortality associated with overweight and obesity is caused chiefly by adverse effects of adiposity on blood pressure, blood lipids, and diabetes,^8^ which increase vascular and renal disease. At any given BMI level, South Asians tend to have substantially more body fat than Europeans (leading to the emergence of the term thin-outside, fat-inside Indian^9^, ^10^, ^11^). Hence, in 2004, WHO considered revising the definition of overweight downwards for South Asians (eg, to 23–27 kg/m^2^), on the basis of the untested assumption that South Asian vascular risks would rise particularly steeply with BMI.^12^

In the Global BMI Mortality Collaboration meta-analyses,^4^ substantial hazards were associated with overweight and obesity in Europe, North America, and East Asia, but not in South Asia^13^ (the opposite of expectations from physiological studies of body fat^14^). But the South Asian studies^5^, ^6^, ^7^ included too few vascular deaths for statistical stability, so a much bigger cohort was needed.^13^

We report the associations between BMI, blood pressure, and cause-specific mortality before age 70 years in a large prospective study of more than half a million adults in the city of Chennai, southern India. In Chennai, underweight is common and obesity uncommon, yet vascular mortality rates are more than double those in the UK or USA, and half of all adult mortality is from cardiac or other vascular causes.^15^

## Methods

### Study design, participants, and procedures

Between Jan 1, 1998, and Dec 31, 2001, adults aged 35 years or older were recruited from the general population of Chennai into a prospective cohort study. Fieldworkers sought to visit all households in two of the ten administrative zones in Chennai city, and all houses in randomly selected streets in five of the other eight zones. Interviewers recorded age, sex, names of family members, socioeconomic status, education, medical history, tobacco smoking, quid chewing, alcohol intake, and diet (whether vegetarian or not). They also measured height, weight, blood pressure, and (in men only, for cultural reasons) waist circumference (appendix pp 3–5). After several minutes' sitting, blood pressure was measured manually twice (once at the beginning of the interview and once at the end), and an average value was calculated. Systolic blood pressure was recorded at the appearance of arterial sounds and diastolic blood pressure at the disappearance (ie, at phase V). Sphygmomanometers were supplied centrally, calibrated daily, and used only by trained fieldworkers. We focused on systolic blood pressure for the main analyses because it was more informative than diastolic blood pressure for prediction of vascular mortality in previous studies.^16^ For training and quality control, 5% of interviewees were selected randomly after each week's fieldwork for prompt re-interview and re-measurement by a fieldwork supervisor. In 2013–14, 10 161 participants from randomly selected streets were resurveyed with the same procedures as at baseline.^17^ The fieldworkers who did the resurveying were blinded to baseline findings. Field methods have been described.^18^

Deaths were identified by linkage to Chennai city mortality records and through active surveillance by household visits from trained graduate non-medical fieldworkers. After the baseline survey, households were visited once in 2002–05, then biennially. To ensure that follow-up visits to the right people occurred, fieldworkers were not given the names of children of participants, and had to ask and record them again. If participants were no longer living there, neighbours were asked when and where they had moved. If participants had moved within the city, attempts were made to visit them. Participants who could not be located on two consecutive biennial visits were marked as lost to active follow-up, but their deaths continued to be sought in city records.

If participants were known or found to have died, a one-page narrative of the death (guided by standard questions about timing of events and any medical attention^15^) was recorded during the household visit. The probable underlying cause of death was then coded by two independent study doctors (to three digits of the 10th edition of the International Classification of Diseases; appendix p 6). Such verbal autopsy methods were necessary because causes of death could not be obtained from city death records.^18^, ^19^, ^20^ A random 5% of narratives were promptly redone for quality control by fieldwork supervisors revisiting houses. Ethics approval for this study was granted by the Oxford Tropical Research Ethics Committee. All patients provided informed consent.

## Statistical analysis

Follow-up was censored at March 31, 2015, emigration from Chennai, or death. The analyses excluded participants aged 80 years or older at baseline, those with chronic disease at entry (myocardial infarction, stroke, cancer, tuberculosis, or asthma; sensitivity analyses additionally exclude diabetes), those with missing data for BMI or systolic blood pressure, and those with implausible or outlying values for BMI (ie, <15 or ≥40 kg/m^2^) or systolic blood pressure (<80 or ≥250 mm Hg). To further limit any effect of pre-existing disease on systolic blood pressure or BMI at baseline, the first 2 years of follow-up were also excluded. Partly because causes of death are less reliable in old age, in the main analyses we focused on deaths in people aged 35–69 years, but did sensitivity analyses for deaths at ages 70–79 years (appendix).

We used Cox regression to relate mortality rate ratios (RRs) to systolic blood pressure and BMI, with adjustment for age at risk (5-year groups), sex, education (seven categories), socioeconomic status (four categories), smoking (ever or never), and weekly or more frequent alcohol intake (ever or never). Associations between systolic blood pressure and mortality were corrected for regression dilution (by categorisation of people by their baseline reading and estimation of the mean usual systolic blood pressure in each category on the basis of the regression dilution ratio—ie, the correlation between resurvey and baseline measurements^17^). They are therefore described as associations between usual systolic blood pressure and mortality. BMI analyses are reported with and without further adjustment for usual systolic blood pressure (which involved dividing the effects of adjustment for baseline systolic blood pressure by the regression dilution ratio).

The mean of the two systolic blood pressure measurements was used in all analyses. It was grouped into four categories (80 to <125 mm Hg, 125 to <145 mm Hg, 145 to <165 mm Hg, and 165 to <250 mm Hg), with RRs calculated relative to the lowest, and plotted against mean usual systolic blood pressure in these baseline-defined categories. BMI was grouped into six categories (15·0 to <18·5 kg/m^2^, 18·5 to <20·0 kg/m^2^, 20·0 to <22·5 kg/m^2^, 22·5 to <25·0 kg/m^2^, 25·0 to <30·0 kg/m^2^, and 30·0 to <40·0 kg/m^2^), and RRs were plotted against mean baseline BMI in these categories. The RR per 10 mm Hg of usual systolic blood pressure was obtained by fitting systolic blood pressure as a continuous variable, with the regression dilution ratio taken as 0·50. We used SAS (version 9.3) for statistical analyses; graphs were plotted with R (version 3.0).

### Role of the funding source

The study funders had no role in study design; data collection, analysis, or interpretation; or writing of the report. All authors had full access to all study data, and share final responsibility for the decision to submit for publication.

## Results

Of the 525 994 adults in Chennai city that we approached to participate in our study, 500 810 were recruited. 25 184 declined to participate. After exclusions (appendix p 7), 414 746 participants aged 35–69 years (mean 46 [SD 9]) remained. 76 672 (18%) had received no formal education (table) and 186 518 (45%) were women (appendix). Among men, 86 517 (38%) smoked and 64 438 (28%) drank (ie, consumed some alcohol in most weeks; table). Among women, however, fewer than 0·1% reported smoking or drinking alcohol (95 female smokers and 106 female drinkers). Overall, mean systolic blood pressure was 127 mm Hg (SD 15) and mean BMI was 23·2 kg/m^2^ (SD 3·8; table). 18 654 (4%) reported previous treatment for hypertension.

**Table:** BMI versus other baseline characteristics in main mortality analyses

		**15·0 to <18·5 (n=36 882)**	**18·5 to <20·0 (n=39 607)**	**20·0 to <22·5 (n=116 045)**	**22·5 to <25·0 (n=110 013)**	**25·0 to <30·0 (n=90 612)**	**30·0 to <40 (n=21 587)**	**Overall (n=414 746)**
Mean BMI, kg/m^2^ (SD)		17·2 (0·9)	19·3 (0·4)	21·3 (0·7)	23·7 (0·7)	26·8 (1·4)	32·5 (2·3)	23·2 (3·8)
Factors strongly affected by adiposity								
	Mean systolic blood pressure, mm Hg (SD)	120 (17)	124 (16)	126 (14)	129 (14)	130 (16)	133 (18)	127 (15)
	Self-reported diabetes	2%	4%	5%	6%	7%	8%	5%
Major potential confounders								
	Median or higher socioecomic status*	36%	42%	50%	59%	66%	68%	55%
	No formal education	28%	25%	21%	17%	14%	12%	18%
	Ever smoker (men only)†	58%	48%	39%	33%	29%	27%	38%
	Ever weekly (or more frequently) alcohol drinker (men only)†	43%	35%	29%	25%	22%	24%	28%

Results are standardised to the age and sex of the 414 746 participants. People with no follow-up at ages 35–69 years, those with pre-existing chronic disease at baseline (ie, heart attack, stroke, tuberculosis, asthma, or cancer), and those with missing or out-of-range BMI or systolic blood pressure were excluded. BMI=body-mass index.

* From housing type and item ownership.

† <0·1% of women in the study smoked or drank.

At baseline, BMI was positively associated with systolic blood pressure (about 1 mm Hg per kg/m^2^) and diabetes, and low BMI was associated with potential confounders, including low socioeconomic status, lack of formal education and, in men, tobacco smoking and alcohol use (table; appendix pp 8–9). At resurvey 14 years later, mean BMI at ages 45–69 years (age-standardised to baseline findings) was 1 kg/m^2^ greater than at baseline, mean systolic blood pressure at these ages was 1 mm Hg greater, and correlations of resurvey and baseline measurements were 0·50 for systolic blood pressure and 0·88 for BMI (appendix pp 10–11). Individual smoking and alcohol use replies (ever or never) were almost identical at baseline and re-survey (appendix p12).

Consistent with the Registrar-General of India's death rates for urban India (appendix pp 13–14), after exclusion of the first 2 years of follow-up, 29 519 deaths at ages 35–69 years (mean 57) were recorded during 4·3 million person-years of subsequent follow-up (mean 11 years [SD 3] per person). 18 082 of the deaths were in men and 11 437 were in women. The cause was vascular in 14 935 deaths (12 504 cardiac, 1881 stroke, and 550 other), cancer in 3323 deaths, renal disease in 2149 deaths, respiratory disease or tuberculosis in 2701 deaths, other specified medical cause in 2832 deaths, unspecified medical cause in 1623 deaths, and external in 1956 deaths.

**Figure 1:**
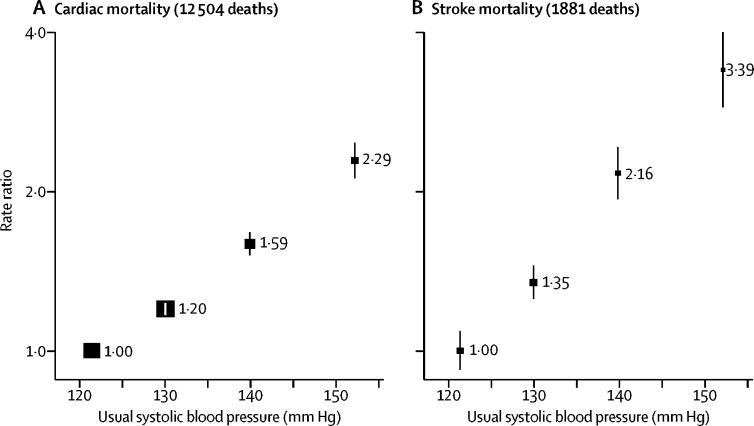
Usual systolic blood pressure *vs* cardiac (A) and stroke (B) mortality at ages 35–69 years in Chennai, India Rate ratios were adjusted for major confounders (age, sex, education, socioeconomic status, smoking, and alcohol) and body-mass index, and omitted the first 2 years of follow-up. Exclusions are as per table 1. For each systolic blood pressure category, the area of the square is inversely proportional to the variance of the category-specific log risk, which also determines the 95% CI (represented by error bars).

Usual systolic blood pressure was strongly positively associated with both cardiac and stroke mortality (figure 1; appendix pp 15–16). These associations were approximately log-linear. In the range 130–160 mm Hg of usual systolic blood pressure, RRs per 20 mm Hg increase in usual systolic blood pressure were 2·45 (95% CI 2·16–2·78) for stroke mortality, 1·74 (1·64–1·84) for cardiac mortality, and 1·84 (1·75–1·94) for all vascular mortality.

**Figure 2:**
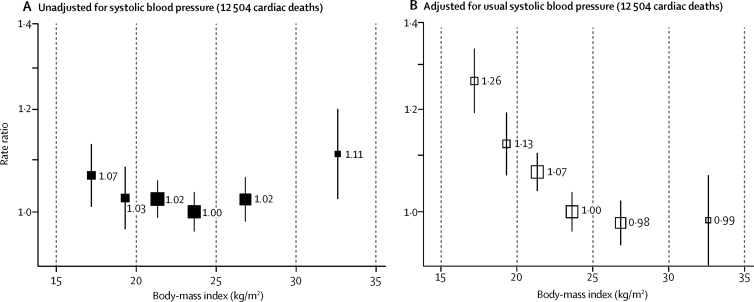
Body-mass index *vs* cardiac mortality at ages 35–69 years in Chennai, India, unadjusted for systolic blood pressure (A), and adjusted for systolic blood pressure (B) Rate ratios were adjusted for major confounders (age, sex, education, socioeconomic status, smoking, and alcohol), and omitted the first 2 years of follow-up. Exclusions are as per table 1. For each body-mass index category, the area of the square is inversely proportional to the variance of the category-specific log risk, which also determines the 95% CI (represented by error bars).

Despite the strong positive association between BMI and systolic blood pressure, and between systolic blood pressure and vascular mortality, BMI was little related to cardiac (figure 2) or stroke (appendix pp 17–19) mortality, with only a slight excess risk at the extremes of BMI. Hence, after adjustment for usual systolic blood pressure, BMI was strongly inversely related to both cardiac and stroke mortality throughout the range 15·0–30·0 kg/m^2^ (ie, the blood-pressure-adjusted vascular mortality was higher in underweight than in overweight participants). When underweight (ie, BMI 15·0–18·5 kg/m^2^) and overweight (ie, BMI 25·0–30·0 kg/m^2^) people were compared, the blood-pressure-adjusted RR was 1·28 (95% CI 1·20–1·38) for cardiac mortality and 1·46 (1·22–1·73) for stroke mortality. BMIs of 30 kg/m^2^ or greater (mean 32·5 kg/m^2^, suggesting WHO grade 1 obesity) were associated with little excess vascular mortality before adjustment for usual systolic blood pressure, and no excess after adjustment (appendix pp 17–19).

**Figure 3:**
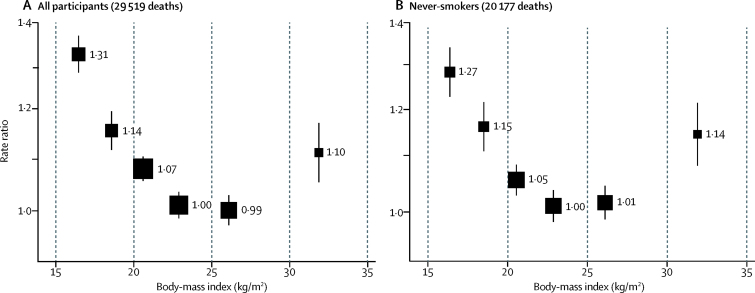
Body-mass index *vs* all-cause mortality at ages 35–69 years in Chennai, India, among all participants (A) and lifelong never-smokers (B) Rate ratios were adjusted for major confounders (age, sex, education, socioeconomic status, alcohol, and smoking [when appropriate]), and omitted the first 2 years of follow-up. Exclusions are as per table 1. For each body-mass index category, the area of the square is inversely proportional to the variance of the category-specific log risk, which also determines the 95% CI (represented by error bars).

Both in all participants and in never-smokers, all-cause mortality was inversely associated with BMI throughout the range 15–30 kg/m^2^ (figure 3). Findings for mortality from selected non-vascular causes are in the appendix (p 17). BMI was little associated with mortality from renal disease, and was inversely associated with cancer mortality (particularly for upper-aerodigestive [chiefly oral] cancer; appendix p 20). BMI was strongly inversely associated with tuberculosis and other respiratory mortality (diseases that can cause weight loss long before death; appendix p 17).

In sensitivity analyses (appendix pp 21–35), the associations of systolic blood pressure and BMI with cardiac and other vascular mortality were similar in both sexes, and not materially changed by excluding the few people with diabetes at baseline (despite twofold RRs for diabetes *vs* vascular and all-cause mortality at age 35–69 years), by excluding a further 3 years of follow-up, or by using usual BMI instead of baseline BMI. Sensitivity analyses of vascular mortality in which people who smoked, drank alcohol, or chewed quids were excluded yielded similar findings, and were little altered by adjustment (or stratification) for socioeconomic status. In sensitivity analyses for the associations of BMI and non-vascular mortality (appendix pp 26–35), the inverse associations between BMI and cancer and respiratory mortality were stronger in men than women. The associations with cancer were somewhat attenuated by excluding smokers, alcohol drinkers, and quid chewers, and those with tuberculosis were attenuated by adjustment for social factors (appendix pp 26–35).

Waist circumference at baseline (available only in men) had a correlation of 0·52 with BMI. Its associations with vascular and all-cause mortality were broadly similar to those for BMI, but not as strong (appendix p 36). Likewise, the associations of diastolic blood pressure with vascular mortality were similar to those of systolic blood pressure (appendix pp 37–38). Mortality showed shallower relationships at age 70–79 years than at age 35–69 years with systolic blood pressure, BMI, and BMI adjusted for usual systolic blood pressure (appendix pp 39–41).

## Discussion

**Figure 4:**
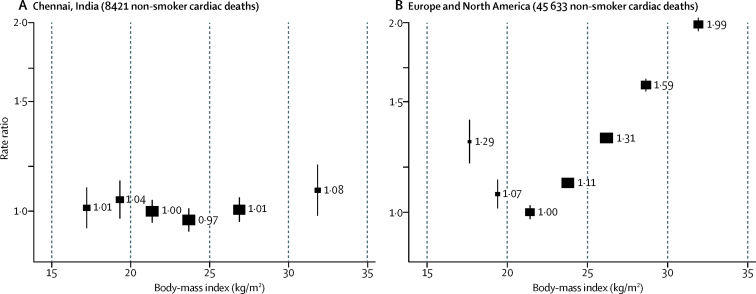
Body-mass index *vs* cardiac mortality among never-smokers in Chennai, India (A) and Europe and North America (B) Rate ratios were adjusted for major confounders (ie, age, sex, social factors, and alcohol in Chennai, and age and sex in Europe and North America), excluding early follow-up (2 years in Chennai, 5 years in Europe and North America), those with pre-existing chronic disease at baseline, and those with body-mass indices <15 kg/m^2^ and >35 kg/m^2^. Estimates for Europe and North America are from the Global BMI Mortality Collaboration.^4^ For each body-mass index category, the area of the square is inversely proportional to the variance of the category-specific log risk, which also determines the 95% CI (represented by error bars).

In this South Asian population, we found little association between BMI and either cardiac or stroke mortality, even though BMI is a strong determinant of blood pressure and diabetes, both of which are strong determinants of vascular mortality. The limited vascular hazards associated with overweight and obesity differ strikingly from the substantial hazards noted in Europe and North America, where prospective studies (if properly analysed to limit the effects of reverse causality) show cardiac mortality to be minimal in people with BMI in the range 20–22·5 kg/m^2^, above which mortality doubles per 10 kg/m^2^ increase in BMI (figure 4; appendix p 42). This contrast is surprising, especially because, at a given BMI, South Asians tend to have more body fat than Europeans.^9^, ^10^, ^11^

Mortality from renal disease was also little associated with BMI, but mortality from other non-vascular causes was inversely associated with BMI across the range 15–30 kg/m^2^, chiefly because of tuberculosis and other respiratory diseases (which are strongly related to poverty, and can be associated with weight loss many years before death) and oral cancer (which is strongly related to quid chewing, a strong correlate of lower BMI [appendix p 8]).

In our study of more than 500 000 adults, we used novel fieldwork methods for large-scale follow-up and verbal autopsy of the causes of any deaths (with biennial home visits to identify deaths and obtain structured narratives for coding of underlying causes^18^). These methods allowed us to assign causes to 70 000 deaths (30 000 deaths before age 70 years, plus another 40 000 at older ages or in people with pre-existing disease), far more than in previous South Asian prospective studies. In addition to large size, however, prospective studies need adequate baseline measurements and reliable record linkage, both of which were achieved in our study.

The BMI and blood pressure measurements at baseline and among 10 000 survivors resurveyed 14 years later (by fieldworkers blinded to baseline data) were correlated about as strongly as expected for measurements 14 years apart,^16^, ^21^ confirming the reliability of both surveys. At resurvey, smoking, drinking, and personal identifiers had changed in fewer than 0·01% of individuals (appendix p12), indicating that these data were reliably recorded at baseline. Although low BMI is associated with smoking, alcohol, and poverty, the main findings for cardiac mortality were little changed by exclusion of everyone who smoked or drank alcohol (or chewed quids). Among the remainder, adjustment or stratification for indices of social factors did not materially change the findings, so finer adjustment for social factors would probably not have done so. Future resurveys will collect supplementary information about socioeconomic status, access to health care, and risk factors such as diet and physical activity, for which limited information was available at baseline.

Causes of death could not be obtained from Chennai city mortality records. Thus, a validated physician-coded verbal autopsy procedure was developed to assign causes.^18^ Almost half the deaths before age 70 years were vascular (chiefly cardiac), consistent with other estimates for urban south India or all of India; 85% of the number of deaths expected at ages 45–69 years from these estimates were identified by the follow-up procedures in this study (appendix p 14).^22^, ^23^ Although blood pressure seemed little associated with non-vascular mortality, it was strongly associated with cardiac mortality and even more so with stroke mortality (both about as steeply as in European and North American populations^16^). These strong relationships with blood pressure show that the reliability of record linkage and of cause-of-death assignment were sufficient to detect any substantial associations between BMI and cardiac or stroke mortality, yet none was found.

Because the relations between BMI and cardiac and stroke mortality were similarly flat, any confusion between these two vascular causes would not have materially affected either relationship. The verbal autopsies did not distinguish between ischaemic and haemorrhagic stroke (which could well have different relationships with BMI), or between different types of heart disease (although most cardiac deaths would have been due directly or indirectly to ischaemic heart disease^23^). Analyses describe only mortality, not incidence (because non-fatal myocardial infarction and stroke records were not available). Previous studies^24^, ^25^ have shown that, although BMI is positively associated with vascular disease incidence in European and North American populations, case-fatality rates can be inversely related to BMI.

The absence of substantial excess vascular mortality with overweight (BMI 25–30 kg/m^2^) and grade 1 obesity matches findings from the only other major prospective studies in India (from Mumbai city^6^ and rural Kerala^7^). Those studies, however, each involved only around 10 000 deaths, and the only other South Asian study^5^ was much smaller. Hence, even meta-analyses^4^, ^13^ of the three previous South Asian prospective studies included too few vascular deaths in participants, particularly among those with obesity, for statistical stability. Our study triples the total number of deaths available. Although the overall evidence available suggests that BMI is not strongly associated with vascular risk in South Asians, it also shows that systolic blood pressure increases with BMI about as strongly in South Asian as in European and North American populations, as does diabetes.^21^ Because systolic blood pressure strongly affects vascular risk, the flat relationship between BMI and vascular mortality means that, at a given level of usual blood pressure, BMI is strongly inversely related to cardiac and stroke mortality.

Hence, some adverse correlate of low BMI (which might act only during development^26^, ^27^ or might still be acting in adult life) is strongly associated with increased vascular mortality in South Asia—or, equivalently, some protective correlate of high BMI is associated with reduced vascular mortality sufficiently strongly to outweigh the adverse effects of high BMI on blood pressure, blood lipids, and diabetes. Unfortunately, BMI (and, in men, waist circumference) was our only measure of adiposity; we had no measurements of fat distribution, body impedance, blood biochemistry, or fitness.

The most immediately actionable finding for individual or population health is confirmation here, as elsewhere, of the importance of blood pressure to vascular mortality. Effective antihypertensive drugs are available as generics, and trials have shown that they safely reduce stroke and myocardial infarction. Among people of a given age, those who would gain most from blood pressure reduction would be those at greatest absolute risk (many of whom are normotensive or hypertensive people who already have some history of vascular disease or diabetes but still have a good quality of life, rather than people with moderate hypertension and no history of disease).

The most surprising finding, however, is the substantial discrepancy between this population and many other populations in the relationship between BMI and vascular mortality. In European and North American populations, the strong association between BMI and risk is accounted for mainly by the effects of aspects of adiposity on blood pressure, diabetes, and dyslipidaemia, which in turn cause vascular disease. In India, the associations between BMI and blood pressure and between BMI and diabetes are about as strong as in Europe or North America, as are the effects of blood pressure and diabetes on vascular mortality. Yet the prospective study evidence now available from South Asia shows reliably that, over quite a wide range, BMI has little association with vascular mortality. This finding points to the existence of novel and important causes of vascular mortality that are closely correlated with below-average BMI, which could well be of some relevance in all populations.
