# Supplementary material for: Body-mass index, blood pressure, and cause-specific mortality in India: a prospective cohort study of 500 810 adults
Source: Lancet Glob Health. 2018 Jun 13;6(7):e787–94. doi: 10.1016/S2214-109X(18)30267-5 (PMC6005121; doi:10.1016/S2214-109X(18)30267-5)
Supplement: Supplementary appendix [file mmc1.pdf]

THE LANCET
Global Health

## Supplementary appendix

This appendix formed part of the original submission and has been peer reviewed. We post it as supplied by the authors.

Supplement to: Gajalakshmi V, Lacey B, Kanimozhi V, Sherliker P, Peto R, Lewington S. Body-mass index, blood pressure, and cause-specific mortality in India: a prospective cohort study of 500 810 adults. *Lancet Glob Health* 2018; **6:** e787–94.

*This page intentionally left blank*

# Body-mass index, blood pressure and cause-specific mortality in India: Prospective study of 500 000 adults

## Supplementary appendix, Table of Contents


| | Page |
|---|---|
| **Questionnaire** | 3-5 |
| **Table S1:** Mortality endpoints and their ICD-10 codes | 6 |
| **Table S2:** Number of participants at baseline and exclusions from mortality analyses, by age and sex | 7 |
| **Table S3:** Baseline characteristics of participants included in the main mortality analyses at ages 35-69 years, by SBP and BMI | 8 |
| **Table S4:** Blood pressure and BMI at baseline, by age and sex | 9 |
| **Table S5:** Baseline characteristics of participants included in the main mortality analyses at ages 35-69 years, among those resurveyed and those not resurveyed | 10 |
| **Table S6:** Blood pressure and BMI of resurveyed participants, by age at baseline and at resurvey | 11 |
| **Table S7:** Smoking and drinking at baseline and at resurvey, among resurveyed men and women | 12 |
| **Table S8:** Baseline characteristics of participants included in the main mortality analyses at ages 35-69 years, by follow-up status | 13 |
| **Table S9:** Observed and expected numbers of deaths at ages 45-69 during follow-up years 3-12 | 14 |
| **Table S10:** Mean baseline values and estimated usual values of SBP and DBP, by age | 15 |
| **Figure S1:** Cause-specific mortality by SBP at ages 35-69 | 16 |
| **Figure S2:** Cause-specific mortality by BMI at ages 35-69 | 17 |
| **Figure S3:** All vascular and all-cause mortality by BMI at ages 35-69 | 18 |
| **Figure S4:** Cause-specific mortality by BMI at ages 35-69, additionally adjusted for usual SBP | 19 |
| **Figure S5:** Cancer mortality by BMI at ages 35-69 | 20 |
| **Figures S6-S10:** Sensitivity analyses for cause-specific mortality, by SBP at ages 35-69 | |
| Excluding diabetics | 21 |
| Excluding ever-smokers, ever-drinkers and quid chewers | 22 |
| Excluding the first 5 years of follow-up | 23 |
| In men only | 24 |
| In women only | 25 |
| **Figures S11-S20:** Sensitivity analyses for cause-specific mortality by BMI at ages 35-69 | |
| Additionally adjusted for quid chewing | 26 |
| Excluding diabetics | 27 |
| Excluding ever-smokers | 28 |
| Excluding ever-smokers, ever-drinkers and quid chewers | 29 |
| Excluding ever-smokers, ever-drinkers and quid chewers, prior to adjustment for social class or education | 30 |
| Restricted to those of lower socioeconomic status | 31 |
| Restricted to those of median or higher socioeconomic status | 32 |
| Excluding the first 5 years of follow-up | 33 |
| In men only | 34 |
| In women only | 35 |
| **Figure S21:** All vascular and all-cause mortality by waist circumference at ages 35-69 | 36 |
| **Figure S22:** Cause-specific mortality by DBP at ages 35-69 | 37 |
| **Figure S23:** Cause-specific mortality by DBP at ages 70-79 | 38 |
| **Figure S24:** Cause-specific mortality by SBP at ages 70-79 | 39 |
| **Figure S25:** Cause-specific mortality by BMI at ages 70-79 | 40 |
| **Figure S26:** Cause-specific mortality by BMI at ages 70-79, additionally adjusted for usual SBP | 41 |
| **Figure S27:** Stroke mortality by BMI in Chennai and in Europe/N.America | 42 |

## English translation of the Tamil-language baseline survey form used in 1998–2001 by fieldworkers visiting all households in randomly chosen streets in Chennai city

City zone (1-10) ☐ ☐ Project ID No. ☐☐☐☐☐

Division No. ☐ ☐ ☐ Result of visit (1-4, from Part 4) ☐

---

### Part 1 - Identification details for the entire household

1. **Full name of the head of the household:**
   and if male, father's name and if female, spouse's name:
2. **Name of the respondent:**
   and relationship to head, if different:
3. **Ration card holder (Y/N)**
   and if Yes, ration card number:
4. **Present residential address:**
   and second address in Chennai, if any:
5. **Religion:**

6. **Mother tongue:**

7. **Profile of all persons in the household** [add extra lines if more than 10 are needed]

| | Name | Age | Sex | Marital status* | Years of education | Relation to head | Any habits?** If Yes, list all | Occu-pation |
|---|---|---|---|---|---|---|---|---|
| 1 | | | | | | | | |
| 2 | | | | | | | | |
| 3 | | | | | | | | |
| 4 | | | | | | | | |
| 5 | | | | | | | | |
| 6 | | | | | | | | |
| 7 | | | | | | | | |
| 8 | | | | | | | | |
| 9 | | | | | | | | |
| 10 | | | | | | | | |

* U=Unmarried, M=Married, W=Widowed, D=Divorced, S=Separated

** SM=Smoking, CH=Chewing areca/betel/tobacco quids, SF=Snuff use, AL=Alcohol drinking

8. **Socio-economic status of the household**

☐ Type of house (1 Individual, 2 Flat, 3 Portion, 4 Hut, 5. Other)
☐ Number of rooms
☐ Separate bathroom (0 No, 1 Yes)
☐ Fuel used for cooking (1 Gas, 2 Wood, 3 Kerosene)
☐ Vehicle (0 None, 1 Car, 2 Two-wheeler, 3 Cycle, 4 Other: specify)
☐ Refrigerator (0 No, 1 Yes)
☐ House (0 Owner, 1 Tenant)
☐ Television (0 No, 1 Only black and white, 2 Colour TV)
☐ Wet grinder (0 No, 1 Yes)

9. **Any cancer in the family (Y/N)? [If more than one, give this information for each case]**

Name: Sex: Relationship to head: Age at onset:

Year cancer diagnosed: Site of cancer origin (if known): Alive/dead?

## Part 2 – MEN. Questions to be asked only to men of age 35 or older

1. **Name:** **Father's name:** **Wife's name:**
2. **Age (years):** **Date of birth:** **Place of birth:**
3. **Height (cm):** **Weight (kg):** **Waist (cm):**
4. **FIRST blood pressure reading (SBP/ DBP, mmHg): ……../ ……..**
5. **Peak expiratory flow rate (L / minute):**
6. **Main occupations**

| | Occupation | Main employer | Describe any significant chemical exposure at work | Age began | Age stopped |
|---|---|---|---|---|---|
| 1 | | | | | |
| 2 | | | | | |

7. **Diet:** ☐Vegetarian, no eggs/ ☐Non-vegetarian/ ☐Vegetarian with eggs; Brahmin diet? (Y/N)
8. **Previous history of treated disease**

| | Y/N | Time since onset | | Y/N | Time since onset |
|---|---|---|---|---|---|
| Hypertension | | | Cancer (& site) | | |
| Asthma | | | Stroke/paralysis | | |
| Diabetes | | | Heart attack | | |
| Tuberculosis | | | Other (specify*) | | |

*Coded later as cardiac/not

9. **Habits – smoking, chewing, snuff-taking, drinking**

| Smoking | 0/blank=never, 1=ex, 2=current | Frequency per day | Age began | Age stopped | Duration (years) |
|---|---|---|---|---|---|
| Bidis | | | | | |
| Cigarettes | | | | | |
| Other (specify) | | | | | |

Circle places you smoke: 1. House, 2. Toilet, 3. Office, 4. Bus Stop, 5. Rly Stn, 6. Street, 7. Other

Time spent per day smoking (minutes): Money spent per month on smoking (Rs):

| Quid chewing or snuff-taking | 0/blank=never, 1=ex, 2=current | Frequency per day | Age began | Age stopped | Duration (years) |
|---|---|---|---|---|---|
| Areca nut (AN) alone | | | | | |
| Tobacco alone | | | | | |
| AN + betel leaf (BL) | | | | | |
| AN + tobacco | | | | | |
| AN + BL + tobacco | | | | | |
| Manufactured quid<br>- containing tobacco | | | | | |
| - without tobacco | | | | | |
| **Snuff-taking** | | | | | |

| Alcohol drinking | 0/blank=never, 1=ex, 2=current | Frequency per week | Amount /event | Age began | Age stopped | Duration (years) |
|---|---|---|---|---|---|---|
| Indian liquor (arrack/toddy) | | | | | | |
| Whisky | | | | | | |
| Beer | | | | | | |
| Brandy | | | | | | |
| Other (specify) | | | | | | |

10. **SECOND blood pressure reading (SBP/DBP, mmHg): ……../ ……..** **[Jump to Part 4]**

## Part 3 – WOMEN. Questions to be asked only to women of age 35 or older

1. **Name:** **Husband's name:**
2. **Age (years):** **Date of birth:** **Place of birth:**
3. **Height (cm):** **Weight (kg):**
4. **FIRST blood pressure reading (SBP/ DBP, mmHg): ……../ ……..**
5. **Peak expiratory flow rate (L / minute):**
6. **Main occupations**

| | Occupation | Main employer | Describe any significant chemical exposure at work | Age began | Age stopped |
|---|---|---|---|---|---|
| 1 | | | | | |
| 2 | | | | | |

7. **Diet:** ☐Vegetarian, no eggs/ ☐Non-vegetarian/ ☐Vegetarian with eggs; Brahmin diet? (Y/N)
8. **Previous history of treated disease**

| | Y/N | Time since onset | | Y/N | Time since onset |
|---|---|---|---|---|---|
| Hypertension | | | Cancer (& site) | | |
| Asthma | | | Stroke/paralysis | | |
| Diabetes | | | Heart attack | | |
| Tuberculosis | | | Other (specify*) | | |

*Coded later as cardiac/not

9. **Habits – smoking, chewing, snuff-taking, drinking**

| | 0/blank=never, 1=ex, 2=current | Frequency per day | Age began | Age stopped | Duration (years) |
|---|---|---|---|---|---|
| **Smoking:** Bidis | | | | | |
| Cigarettes | | | | | |
| Other (specify) | | | | | |
| **Chewing:** AN alone | | | | | |
| Tobacco alone | | | | | |
| AN + betel leaf (BL) | | | | | |
| AN + tobacco | | | | | |
| AN + BL + tobacco | | | | | |
| Manufactured quid - containing tobacco | | | | | |
| - without tobacco | | | | | |
| **Snuff-taking** | | | | | |
| **Alcohol** (specify type, frequency & quantity) | | | | | |

10. **SECOND blood pressure reading (SBP/DBP, mmHg): ……../ ……..**

## Part 4: Final result of visit(s) to this household

☐ 1=Complete information, 2=Partial information, 3=No adult ever available / house locked, etc
4=Interview refused – give reason(s):

☐ No. of visits undertaken, and date of visit 1: of visit 2: of visit 3:

**Interviewed by:** **Date of final interview:**

**Table S1: Mortality endpoints and their ICD-10 codes**

| Endpoint name | ICD-10 codes |
|---|---|
| VASCULAR | I00-I99 |
| Cardiac | I20-I25 |
| Stroke | I60-I61, I63-I64 |
| Other | Remainder of vascular |
| RENAL | N00-N29 |
| RESPIRATORY | J00-99, A15-A16 |
| Respiratory TB | A15-A16 |
| Other respiratory | Remainder of respiratory |
| CANCER | C00-C97, D00-D48 |
| Upper aerodigestive | C00-C15, C32 |
| Stomach | C16 |
| Lung | C33-C34 |
| Breast | C50 |
| Cervical | C53 |
| Other | Remainder of cancer |
| Other or unspecified medical | Remainder of A00-Y89, excluding external or ill-defined causes |
| External | S00-S99, T00-T98, V01-V99, W00-W99, X00-X99, Y00-Y98, Z00-Z99 |
| Ill-defined causes | R00-R99 (and not standard ICD codes) |
| ALL CAUSES | A00-Y89 |

**Table S2: Number of participants at baseline and exclusions from mortality analyses, by age and sex**

| | Men, by baseline age | | | | Women, by baseline age | | | | Both sexes, by baseline age | |
|---|---|---|---|---|---|---|---|---|---|---|
| | **35-59** | **60-69** | **70-79** | ≥80 (excluded) | **35-59** | **60-69** | **70-79** | ≥80 (excluded) | **35-69** | **70-79** |
| **Initial number of participants** | 216410 | 31640 | 13533 | 3263 | 186363 | 33074 | 12746 | 3781 | 467487 | 26279 |
| **Excluded due to:*** | | | | | | | | | | |
| Prior TB, stroke, heart attack, cancer or asthma | 3455 | 1503 | 726 | 162 | 2993 | 927 | 406 | 110 | 8878 | 1132 |
| SBP or BMI missing | 11025 | 1743 | 889 | 311 | 21883 | 4196 | 1882 | 806 | 38847 | 2771 |
| BMI < 15 | 1504 | 510 | 408 | 144 | 1770 | 607 | 363 | 165 | 4391 | 771 |
| BMI ≥ 40 | 33 | 5 | 1 | 3 | 364 | 71 | 16 | 6 | 473 | 17 |
| SBP < 80 | 35 | 6 | 2 | 2 | 87 | 16 | 9 | 9 | 144 | 11 |
| SBP ≥ 250 | 2 | 1 | 0 | 0 | 3 | 2 | 0 | 0 | 8 | 0 |
| **Total in main analyses** | **200356** | **27872** | **11507** | 0 | **159263** | **27255** | **10070** | 0 | **414746** | **21577** |
| *Total in analyses involving waist circumference* | *196517* | *27319* | *11252* | *0* | *0* | *0* | *0* | *0* | *223836* | *11252* |

BMI=body-mass index, SBP=systolic blood pressure

*Numbers are for progressive exclusion of participants.

**Table S3: Baseline characteristics of the participants* included in the main mortality analyses at ages 35-69 years, by SBP and BMI**

| | Baseline SBP, mmHg | | | | Baseline BMI, kg/m$^2$ | | | | | | All |
|---|---|---|---|---|---|---|---|---|---|---|---|
| | 80-<125 | 125-<144 | 145-<165 | 165-<250 | 15-<18.5 | 18.5-<20 | 20-<22.5 | 22.5-<25 | 25-<30 | 30-<40 | |
| Number of participants | 189 814 | 180 901 | 34 548 | 9 483 | 36 882 | 39 607 | 116 045 | 110 013 | 90 612 | 21 587 | 414 746 |
| Female, % | 49% | 39% | 53% | 63% | 45% | 40% | 36% | 41% | 57% | 74% | 45% |
| Mean (SD) age, years | 44 (9) | 47 (9) | 53 (9) | 55 (9) | 47 (10) | 47 (10) | 46 (9) | 46 (9) | 46 (9) | 46 (9) | 46 (9) |
| Mean (SD) SBP, mmHg | 115 (8) | 133 (5) | 152 (6) | 178 (12) | 120 (17) | 124 (16) | 126 (14) | 129 (14) | 130 (16) | 133 (18) | 127 (15) |
| Mean (SD) DBP, mmHg | 77 (7) | 86 (7) | 94 (10) | 102 (14) | 78 (10) | 81 (10) | 82 (9) | 84 (9) | 85 (10) | 87 (11) | 83 (10) |
| Mean (SD) BMI, kg/m$^2$ | 22.4 (3.7) | 23.7 (3.6) | 24.7 (4.2) | 24.8 (4.5) | 17.2 (0.9) | 19.3 (0.4) | 21.3 (0.7) | 23.7 (0.7) | 26.8 (1.4) | 32.5 (2.3) | 23.2 (3.8) |
| Mean (SD) height (men), cm | 164 (6) | 164 (5) | 164 (6) | 164 (6) | 164 (6) | 164 (6) | 164 (5) | 164 (5) | 163 (6) | 162 (8) | 164 (6) |
| Mean (SD) height (women), cm | 153 (6) | 154 (6) | 153 (6) | 153 (6) | 153 (7) | 154 (7) | 154 (6) | 154 (6) | 152 (6) | 151 (6) | 153 (6) |
| Median or higher socioeconomic status, %† | 52% | 56% | 60% | 59% | 36% | 42% | 50% | 59% | 66% | 68% | 55% |
| No formal education, % | 20% | 18% | 17% | 16% | 28% | 25% | 21% | 17% | 14% | 12% | 19% |
| Self-reported diabetes, % | 4% | 5% | 9% | 11% | 2% | 4% | 5% | 6% | 7% | 8% | 5% |
| Ever ≥weekly alcohol drinker (men only‡), % | 30% | 27% | 30% | 35% | 43% | 35% | 29% | 25% | 22% | 24% | 28% |
| Ever smoker (men only‡), % | 41% | 36% | 36% | 39% | 58% | 48% | 39% | 33% | 29% | 27% | 38% |
| Quid chewing (men), % | 7% | 6% | 7% | 9% | 9% | 7% | 6% | 6% | 6% | 7% | 6% |
| Quid chewing (women), % | 12% | 10% | 11% | 11% | 17% | 15% | 12% | 10% | 8% | 9% | 11% |
| Vegetarian diet, % | 5% | 4% | 4% | 3% | 4% | 4% | 4% | 5% | 5% | 6% | 4% |
| Mean waist circumference (men only‡), cm | 82 (9) | 84 (9) | 86 (10) | 87 (10) | 74 (8) | 78 (7) | 82 (7) | 85 (7) | 89 (9) | 94 (12) | 83 (9) |
| Previous treatment for hypertension, % | 2% | 4% | 14% | 24% | 2% | 3% | 3% | 5% | 7% | 10% | 5% |

SBP= systolic blood pressure, BMI= body-mass index.

* Results are standardised to the age and sex at entry of the 414 746 participants, after exclusions as in Table S2.

† Socioeconomic status was measured using a composite score of housing type and ownership of various material goods (eg, refrigerator).

‡ <0.1% of women reported ever smoking or ever drinking alcohol at least weekly; waist circumference was measured in men only.

## Table S4: Blood pressure and BMI at baseline, by age and sex*

| Age at baseline, years | Participants | Mean age, years | Mean SBP, mmHg | Mean DBP†, mmHg | Mean BMI, kg/m$^2$ |
|---|---|---|---|---|---|
| **Men** | | | | | |
| 35 - 39 | 71165 | 36 | 124 | 82 | 22.5 |
| 40 - 44 | 44390 | 41 | 126 | 83 | 22.7 |
| 45 - 49 | 37402 | 46 | 128 | 84 | 22.8 |
| 50 - 54 | 27991 | 51 | 130 | 85 | 22.8 |
| 55 - 59 | 19408 | 56 | 132 | 85 | 22.6 |
| 60 - 64 | 16758 | 61 | 134 | 85 | 22.4 |
| 65 - 69 | 11114 | 66 | 135 | 85 | 22.1 |
| 70 - 74 | 7562 | 71 | 137 | 85 | 21.7 |
| 75 - 79 | 3945 | 76 | 137 | 85 | 21.3 |
| **Women** | | | | | |
| 35 - 39 | 51297 | 36 | 121 | 80 | 23.8 |
| 40 - 44 | 35150 | 41 | 124 | 82 | 24.2 |
| 45 - 49 | 30783 | 46 | 127 | 83 | 24.1 |
| 50 - 54 | 23503 | 51 | 130 | 84 | 24.1 |
| 55 - 59 | 18530 | 56 | 133 | 85 | 23.8 |
| 60 - 64 | 16041 | 61 | 135 | 85 | 23.4 |
| 65 - 69 | 11214 | 66 | 137 | 85 | 23.0 |
| 70 - 74 | 6965 | 71 | 139 | 85 | 22.5 |
| 75 - 79 | 3105 | 76 | 140 | 85 | 22.2 |
| **Both sexes** | | | | | |
| 35-69 | 414746 | 46 | 127 | 83 | 23.2 |
| 70-79 | 21577 | 72 | 138 | 85 | 21.9 |

BMI= body-mass index, SBP=systolic blood pressure , DBP=diastolic blood pressure

* Exclusions as in Table S2.

† Further excludes people with missing or out of range DBP (<40 or ≥150 mmHg; n=28)

**Table S5: Baseline characteristics of the participants* included in the main mortality analyses at ages 35-69 years, among those resurveyed and those not resurveyed**

| | Resurveyed | Not resurveyed |
|---|---|---|
| Number of participants | 9 896 | 404 850 |
| Female, % | 29% | 45% |
| Mean (SD) age, years | 45 (8) | 46 (9) |
| Mean (SD) SBP, mmHg | 127 (14) | 127 (16) |
| Mean (SD) DBP, mmHg | 83 (9) | 83 (10) |
| Mean (SD) BMI, kg/m2 | 23.1 (3.6) | 23.2 (3.8) |
| Mean (SD) height (men), cm | 164 (5) | 164 (6) |
| Mean (SD) height (women), cm | 152 (6) | 153 (6) |
| Median or higher socioeconomic status, %† | 53% | 55% |
| No formal education, % | 24% | 18% |
| Self-reported diabetes, % | 5% | 5% |
| Ever ≥weekly alcohol drinker (men only‡), % | 30% | 28% |
| Ever smoker (men only‡), % | 35% | 38% |
| Quid chewing (men), % | 7% | 6% |
| Quid chewing (women), % | 16% | 11% |
| Vegetarian diet, % | 3% | 4% |
| Mean waist circumference (men only‡), cm | 83 (9) | 83 (9) |
| Previous treatment for hypertension, % | 4% | 5% |

SBP= systolic blood pressure, BMI= body-mass index.
Means and proportions standardised to baseline distributions of age and sex.
* Exclusions as in Table S2.
† Socioeconomic status was measured using a composite score of housing type and ownership of various material goods (eg, refrigerator).
‡ <0.1% of women reported ever smoking or ever drinking alcohol at least weekly; waist circumference was measured in men only.

**Table S6: Blood pressure and BMI of resurveyed participants,* by age at baseline and at resurvey**

**(A) Baseline blood pressure and BMI, by age at baseline**

| Age at baseline, years | Participants | Mean age, years | Mean SBP, mmHg | Mean DBP†, mmHg | Mean BMI, kg/m$^2$ |
|---|---|---|---|---|---|
| 35 - 39 | 3266 | 36 | 123 | 81 | 22.8 |
| 40 - 44 | 2033 | 41 | 125 | 83 | 23.1 |
| 45 - 49 | 1774 | 46 | 126 | 83 | 22.9 |
| 50 - 54 | 1284 | 51 | 130 | 85 | 23.1 |
| 55 - 59 | 812 | 56 | 132 | 85 | 23.1 |
| 60 - 64 | 556 | 61 | 134 | 85 | 23.1 |
| 65 - 69 | 171 | 65 | 132 | 84 | 22.3 |
| 70 - 74 | - | - | - | - | - |
| 75 - 79 | - | - | - | - | - |
| *45-69‡* | *4597* | *54* | *130* | *84* | *23.0* |
| **All** | 9896 | 45 | 126 | 83 | 22.9 |

**(B) Resurvey blood pressure and BMI, by age at resurvey**

| Age at resurvey, years | Participants | Mean age, years | Mean SBP, mmHg | Mean DBP†, mmHg | Mean BMI, kg/m$^2$ |
|---|---|---|---|---|---|
| 35 - 39 | - | - | - | - | - |
| 40 - 44 | - | - | - | - | - |
| 45 - 49 | 1274 | 48 | 130 | 83 | 23.6 |
| 50 - 54 | 2785 | 52 | 130 | 82 | 24.0 |
| 55 - 59 | 1896 | 57 | 132 | 82 | 24.1 |
| 60 - 64 | 1649 | 62 | 133 | 82 | 23.9 |
| 65 - 69 | 1044 | 67 | 135 | 82 | 24.0 |
| 70 - 74 | 747 | 72 | 135 | 82 | 23.7 |
| 75 - 79 | 501 | 77 | 137 | 81 | 23.6 |
| *45-69‡* | *8648* | *55* | *131* | *82* | *23.9* |
| **All** | 9896 | 58 | 132 | 82 | 23.9 |

BMI= body-mass index, SBP=systolic blood pressure, DBP=diastolic blood pressure.

*Exclusion as in Table S2, with further exclusion of those with missing or out of range SBP (<80 or ≥250 mmHg) or BMI (<15 or ≥40) at resurvey.

† Further excludes people with missing or out of range DBP (<40 or ≥150 mmHg) at resurvey (n=1).

‡ Standardised to the age at entry of participants included in the main mortality analyses (eTable 1).

**Table S7: Smoking and drinking at baseline and at resurvey, among resurveyed men and women***

**(A) Smoking status in resurveyed men**

| Smoking status at resurvey | Smoking status at baseline, number of participants | | |
|---|---|---|---|
| | Ever | Never | All |
| Ever | 2507 | 7 | 2514 |
| Never | 0 | 4570 | 4570 |
| All | 2507 | 4577 | 7084 |

**(B) Drinking status† in resurveyed men**

| Drinking status at resurvey | Drinking status at baseline (number of participants) | | |
|---|---|---|---|
| | Ever | Never | All |
| Ever | 2163 | 45 | 2208 |
| Never | 0 | 4876 | 4876 |
| All | 2163 | 4921 | 7084 |

**(C) Smoking status in resurveyed women**

| Smoking status at resurvey | Smoking status at baseline, number of participants | | |
|---|---|---|---|
| | Ever | Never | All |
| Ever | 1 | 0 | 1 |
| Never | 0 | 2970 | 2970 |
| All | 1 | 2970 | 2971 |

**(D) Drinking status† in resurveyed women**

| Drinking status at resurvey | Drinking status at baseline, number of participants | | |
|---|---|---|---|
| | Ever | Never | All |
| Ever | 0 | 0 | 0 |
| Never | 0 | 2971 | 2971 |
| All | 0 | 2971 | 2971 |

* Exclusion as in Table S2.

† Ever or never ≥weekly alcohol drinker

**Table S8: Baseline characteristics of the participants* included in the main mortality analyses at ages 35-69 years, by follow-up status at censoring date (31 March 2015)**

| | Active follow-up | Lost to active follow-up | Dead |
|---|---|---|---|
| Number of participants | 145 315 | 226 210 | 43 221 |
| Female, % | 48% | 44% | 35% |
| Mean (SD) age, years | 45 (9) | 45 (9) | 54 (10) |
| Mean (SD) SBP, mmHg | 127 (15) | 127 (15) | 129 (19) |
| Mean (SD) DBP, mmHg | 83 (9) | 83 (9) | 84 (11) |
| Mean (SD) BMI, kg/m2 | 23.4 (3.8) | 23.1 (3.7) | 22.8 (3.9) |
| Mean (SD) height (men), cm | 164 (5) | 164 (5) | 163 (6) |
| Mean (SD) height (women), cm | 153 (6) | 153 (6) | 152 (7) |
| Median or higher socioeconomic status, %† | 61% | 52% | 47% |
| No formal education, % | 19% | 17% | 26% |
| Self-reported diabetes, % | 5% | 5% | 9% |
| Ever ≥weekly alcohol drinker (men only‡), % | 26% | 27% | 45% |
| Ever smoker (men only‡), % | 33% | 38% | 52% |
| Quid chewing (men), % | 6% | 6% | 8% |
| Quid chewing (women), % | 12% | 10% | 17% |
| Vegetarian diet, % | 4% | 5% | 3% |
| Mean waist circumference (men only‡), cm | 83 (9) | 83 (9) | 82 (9) |
| Previous treatment for hypertension, % | 4% | 4% | 6% |

SBP= systolic blood pressure, BMI= body-mass index.
Means and proportions standardised to baseline distributions of age and sex.
* Exclusions as in Table S2.
† Socioeconomic status was measured using a composite score of housing type and ownership of various material goods (eg, refrigerator).
‡ <0.1% of women reported ever smoking or ever drinking alcohol at least weekly; waist circumference was measured in men only.

**Table S9: Observed and expected* numbers of deaths at ages 45-69 during follow-up years 3-12**

| Age group, years | Follow-up year | | | | | | | | | | |
|---|---|---|---|---|---|---|---|---|---|---|---|
| | 3 | 4 | 5 | 6 | 7 | 8 | 9 | 10 | 11 | 12 | All (3-12) |
| **Expected (E)** | | | | | | | | | | | |
| 45-49 | 289 | 328 | 319 | 355 | 370 | 435 | 420 | 481 | 553 | 503 | 4053 |
| 50-54 | 405 | 388 | 410 | 446 | 450 | 418 | 474 | 462 | 516 | 538 | 4506 |
| 55-59 | 373 | 439 | 426 | 496 | 503 | 584 | 561 | 592 | 647 | 654 | 5275 |
| 60-64 | 541 | 514 | 554 | 536 | 565 | 534 | 628 | 609 | 714 | 723 | 5919 |
| 65-69 | 612 | 664 | 652 | 799 | 789 | 873 | 827 | 893 | 884 | 936 | 7928 |
| All (45-69) | 2220 | 2334 | 2361 | 2632 | 2677 | 2844 | 2910 | 3036 | 3315 | 3353 | 27682 |
| **Observed (O)** | | | | | | | | | | | |
| 45-49 | 266 | 313 | 333 | 301 | 304 | 349 | 325 | 362 | 341 | 309 | 3203 |
| 50-54 | 385 | 412 | 467 | 361 | 416 | 382 | 386 | 370 | 358 | 375 | 3912 |
| 55-59 | 392 | 485 | 479 | 450 | 425 | 579 | 501 | 521 | 466 | 446 | 4744 |
| 60-64 | 559 | 594 | 666 | 504 | 567 | 474 | 504 | 527 | 491 | 505 | 5391 |
| 65-69 | 523 | 615 | 610 | 710 | 711 | 730 | 700 | 702 | 545 | 539 | 6385 |
| All (45-69) | 2125 | 2419 | 2555 | 2326 | 2423 | 2514 | 2416 | 2482 | 2201 | 2174 | 23635 |
| **O-E** | | | | | | | | | | | |
| 45-49 | -23 | -15 | 14 | -54 | -66 | -86 | -95 | -119 | -212 | -194 | -850 |
| 50-54 | -20 | 24 | 57 | -85 | -34 | -36 | -88 | -92 | -158 | -163 | -594 |
| 55-59 | 19 | 46 | 53 | -46 | -78 | -5 | -60 | -71 | -181 | -208 | -531 |
| 60-64 | 18 | 80 | 112 | -32 | 2 | -60 | -124 | -82 | -223 | -218 | -528 |
| 65-69 | -89 | -49 | -42 | -89 | -78 | -143 | -127 | -191 | -339 | -397 | -1543 |
| All (45-69) | -95 | 85 | 194 | -306 | -254 | -330 | -494 | -554 | -1114 | -1179 | -4047 |
| **O/E** | | | | | | | | | | | |
| 45-49 | 0.92 | 0.95 | 1.04 | 0.85 | 0.82 | 0.80 | 0.77 | 0.75 | 0.62 | 0.61 | 0.79 |
| 50-54 | 0.95 | 1.06 | 1.14 | 0.81 | 0.92 | 0.91 | 0.81 | 0.80 | 0.69 | 0.70 | 0.87 |
| 55-59 | 1.05 | 1.10 | 1.13 | 0.91 | 0.85 | 0.99 | 0.89 | 0.88 | 0.72 | 0.68 | 0.90 |
| 60-64 | 1.03 | 1.16 | 1.20 | 0.94 | 1.00 | 0.89 | 0.80 | 0.87 | 0.69 | 0.70 | 0.91 |
| 65-69 | 0.86 | 0.93 | 0.94 | 0.89 | 0.90 | 0.84 | 0.85 | 0.79 | 0.62 | 0.58 | 0.81 |
| All (45-69) | 0.96 | 1.04 | 1.08 | 0.88 | 0.91 | 0.88 | 0.83 | 0.82 | 0.66 | 0.65 | 0.85 |

*The number of deaths observed in the Chennai prospective study (with exclusions as in eTable 1) were compared to those expected given the Registrar-General of India's death rates for urban Tamil Nadu, 2015.

## Table S10: Mean baseline values and estimated usual values of SBP and DBP, by age*

### (A) SBP

| Baseline SBP categories, mmHg | Age 35-69 at baseline | | | Age 70-79 at baseline | | |
|---|---|---|---|---|---|---|
| | Participants | Mean SBP at baseline, mmHg | Estimated usual SBP†, mmHg | Participants | Mean SBP at baseline, mmHg | Estimated usual SBP†, mmHg |
| 80-124 | 188208 | 115.2 | 121.3 | 5496 | 113.6 | 125.8 |
| 125-144 | 179076 | 132.7 | 130.0 | 7904 | 135.2 | 136.6 |
| 145-164 | 33872 | 152.8 | 140.1 | 4750 | 153.8 | 145.9 |
| 165-249 | 9205 | 177.8 | 152.6 | 1966 | 178.7 | 158.3 |
| **All** | 410361 | 127.4 | 127.4 | 20116 | 137.9 | 137.9 |

### (B) DBP‡

| Baseline DBP categories, mmHg | Age 35-69 at baseline | | | Age 70-79 at baseline | | |
|---|---|---|---|---|---|---|
| | Participants | Mean DBP at baseline, mmHg | Estimated usual DBP†, mmHg | Participants | Mean DBP at baseline, mmHg | Estimated usual DBP†, mmHg |
| 40-74 | 66855 | 69.0 | 78.1 | 3845 | 68.3 | 79.1 |
| 75-84 | 180270 | 80.5 | 82.2 | 6074 | 80.2 | 83.3 |
| 85-94 | 125806 | 88.8 | 85.1 | 6680 | 89.4 | 86.5 |
| 95-149 | 37407 | 101.7 | 89.6 | 3513 | 102.7 | 91.1 |
| **All** | 410338 | 83.1 | 83.1 | 20112 | 84.9 | 84.9 |

SBP=systolic blood pressure, DBP=diastolic blood pressure.

*Exclusions as in Table S2, with the additional exclusion of participants with less than 2 years of follow-up.

† At baseline survey, blood pressure was measured twice (first with strong digit preference, then with a slower mercury fall past the first value) and averaged. Usual SBP and usual DBP were estimated by correcting mean SBP and mean DBP at baseline for regression dilution ratios of 0.50 and 0.35, respectively (self-correlations [Pearson] between baseline and resurvey blood pressure values in resurvey participants; further adjustment for age at baseline and sex did not materially change these self-correlations).

‡ Further excludes participants with out of range DBP (<40 or ≥150 mmHg) at baseline (n=27)

# Figure S1: Cause−specific mortality by SBP at ages 35−69

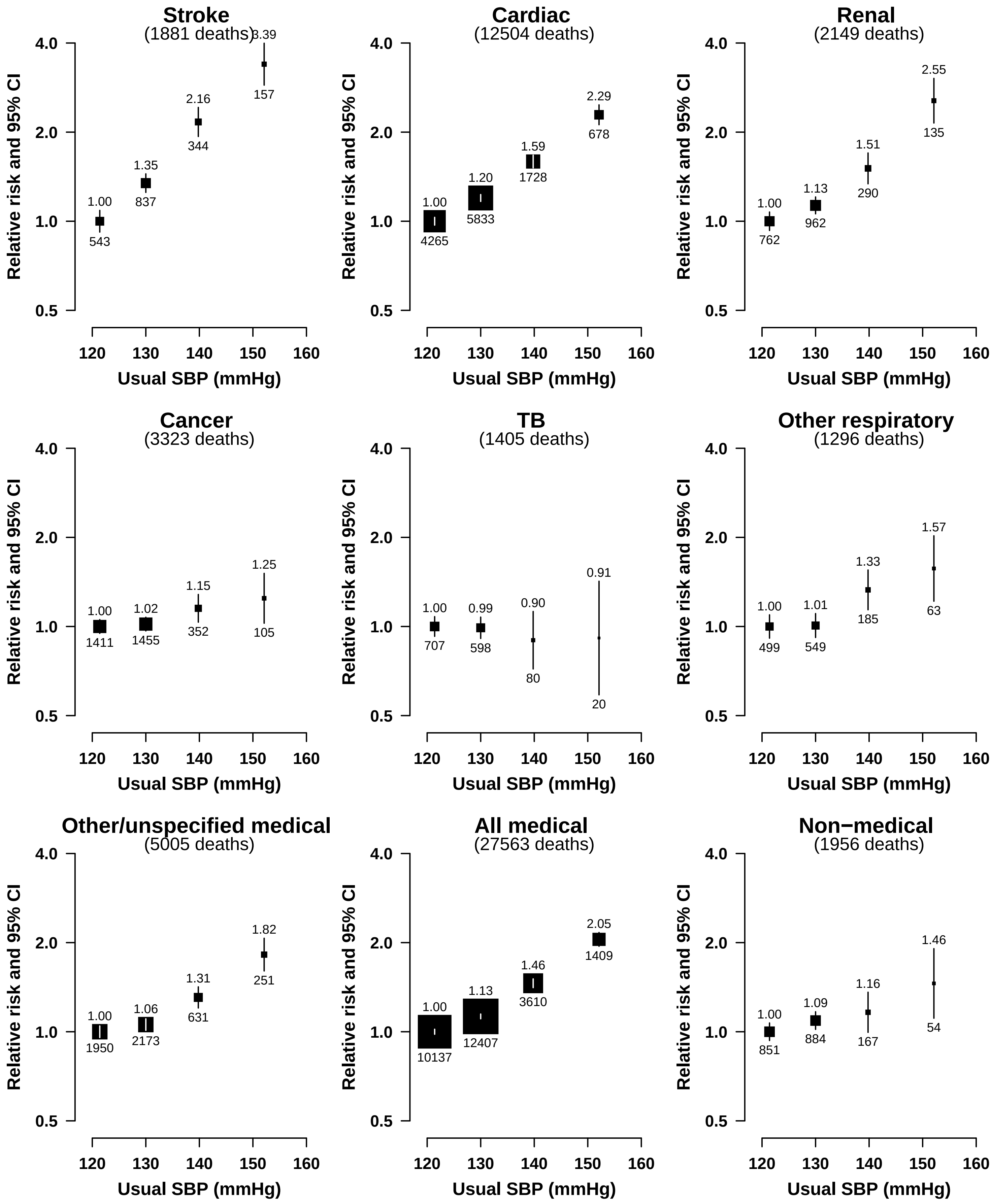


SBP=systolic blood pressure. Death rate ratios (RR) at ages 35−69 years are adjusted for age, sex, education, socioeconomic status, smoking, alcohol and BMI. For each category, area of the square is inversely proportional to the variance of the category−specific log risk, which also determines the confidence interval (CI). RR shown above each square and the number of events below. Exclusions as in Table S2, with further exclusion of the first 2 years of follow−up.

## Figure S2: Cause−specific mortality by BMI at ages 35−69

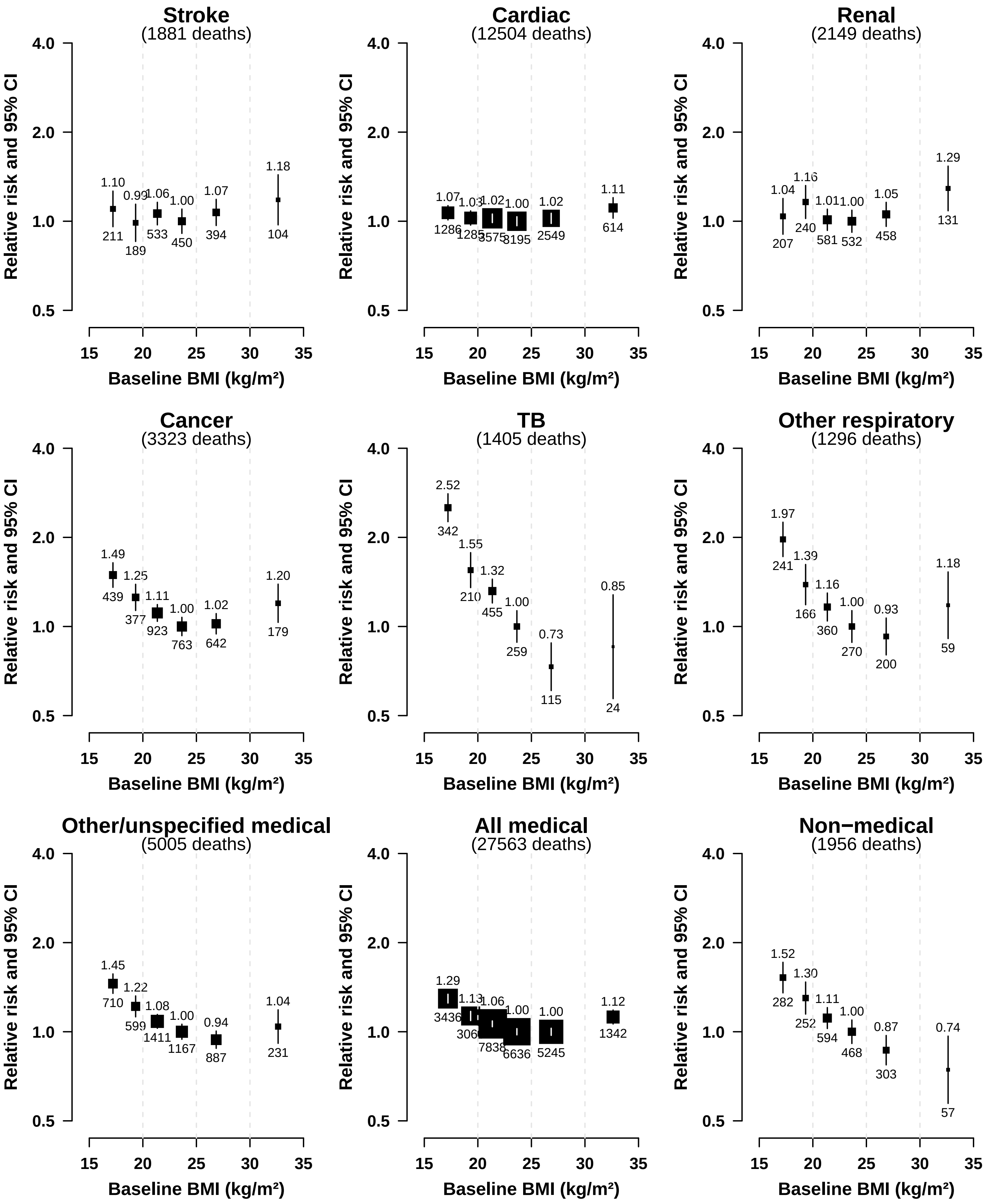


BMI=body−mass index. Death rate ratios (RR) at ages 35−69 years are adjusted for age, sex, education, socioeconomic status, smoking and alcohol. BMI categories: 15−18.5, 18.5−20, 20−22.5, 22.5−25 (reference), 25−30, 30−40 kg/m². For each category, area of the square is inversely proportional to the variance of the category−specific log risk, which also determines the confidence interval (CI). RR shown above each square and the number of events below. Exclusions as in Table S2, with further exclusion of the first 2 years of follow−up.

## Figure S3: All vascular and all−cause mortality at ages 35−69 years by baseline BMI, before and after adjustment for usual SBP

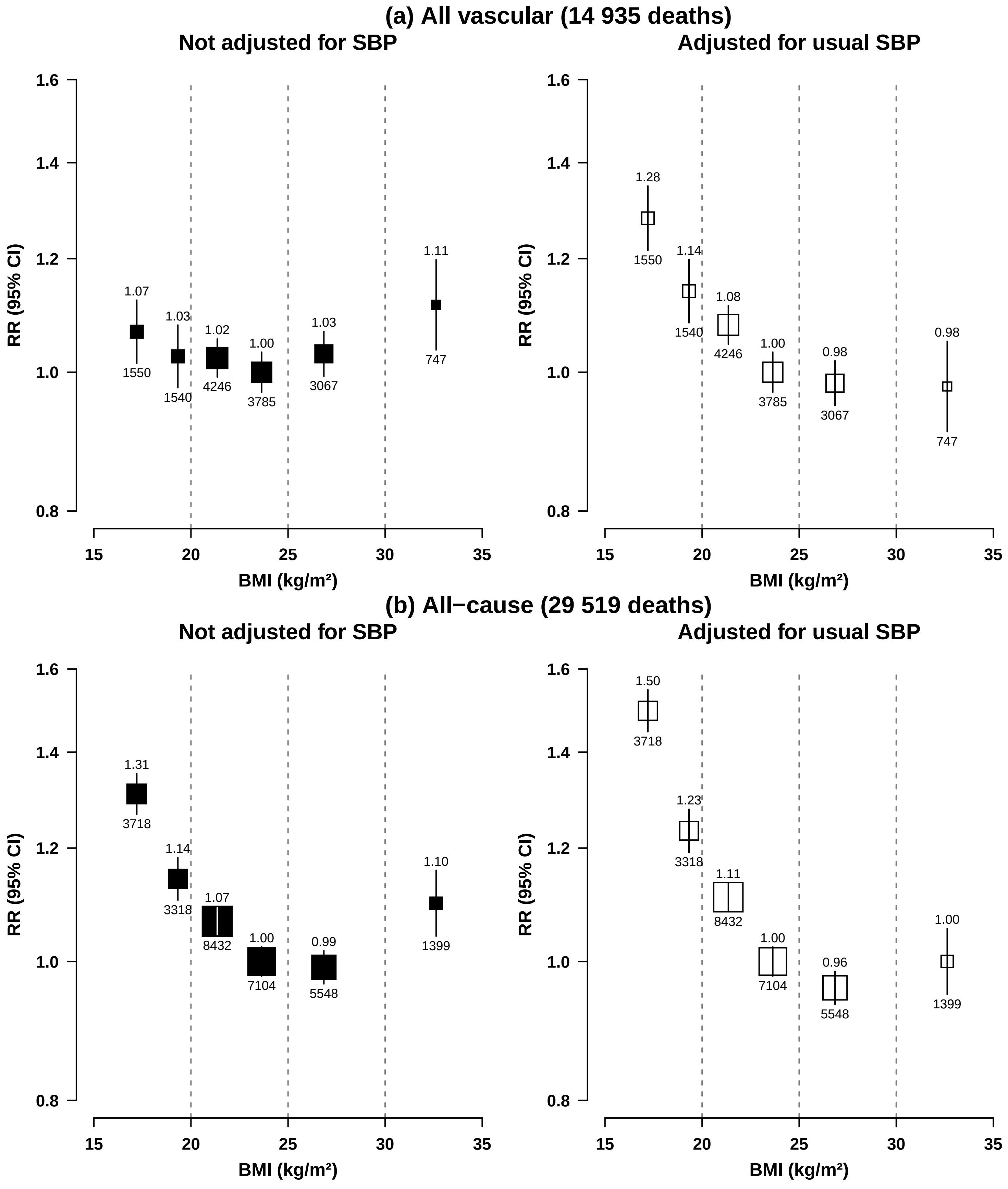


Death rate ratios (RR) at ages 35−69 years are adjusted for age, sex, education, socioeconomic status, smoking and alcohol, with further adjustment for usual SBP where indicated. Exclusions as in Table S2, with further exclusion of the first 2 years of follow-up.

# Figure S4: Cause−specific mortality by BMI at ages 35−69 additionally adjusted for usual SBP

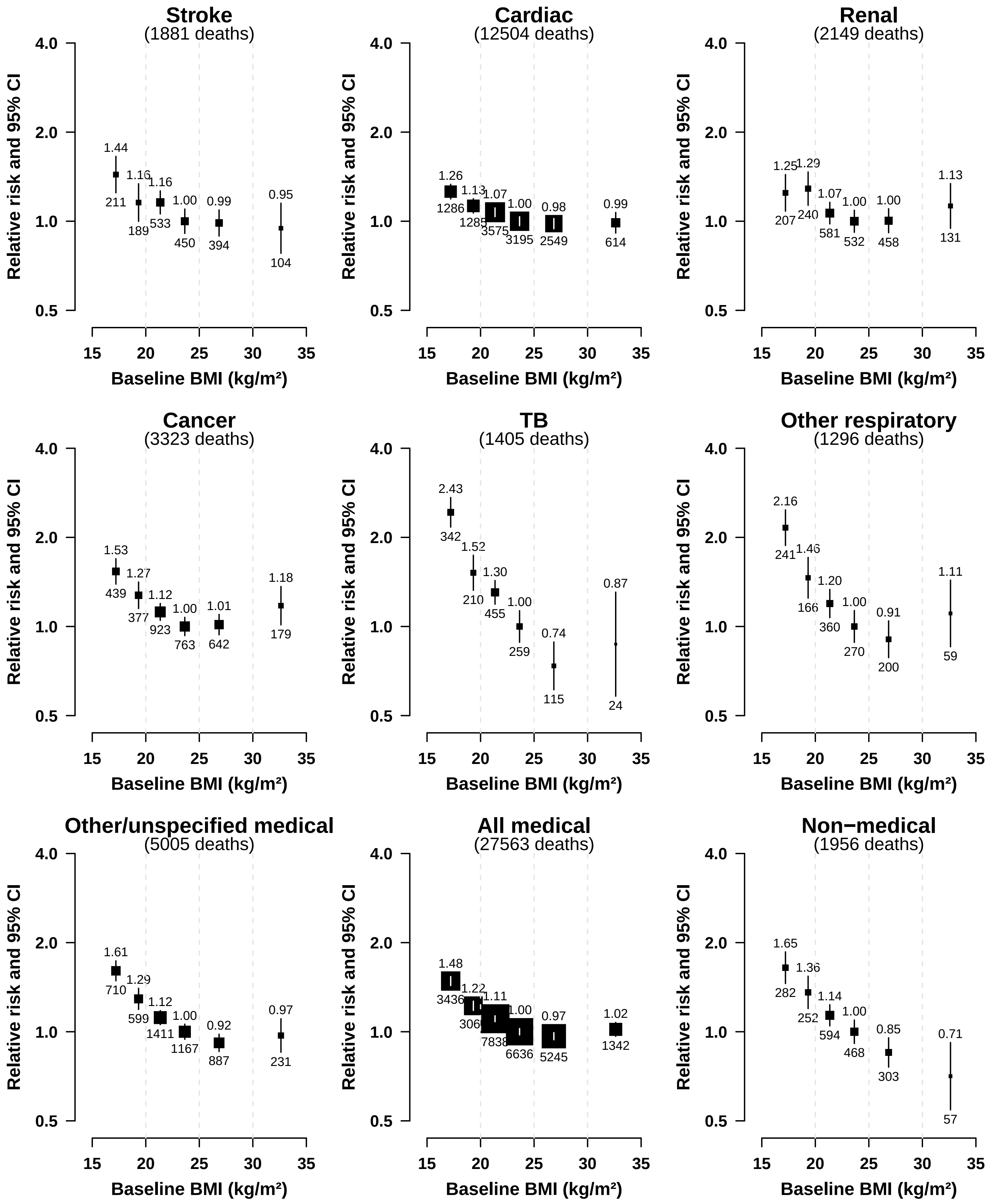


BMI=body−mass index. Death rate ratios (RR) at ages 35−69 years are adjusted for age, sex, education, socioeconomic status, smoking and alcohol. BMI categories: 15−18.5, 18.5−20, 20−22.5, 22.5−25 (reference), 25−30, 30−40 kg/m². For each category, area of the square is inversely proportional to the variance of the category−specific log risk, which also determines the confidence interval (CI). RR shown above each square and the number of events below. Exclusions as in Table S2, with further exclusion of the first 2 years of follow−up.
ADDITIONALLY ADJUSTED FOR USUAL SBP

# Figure S5: Cancer mortality by BMI at ages 35−69

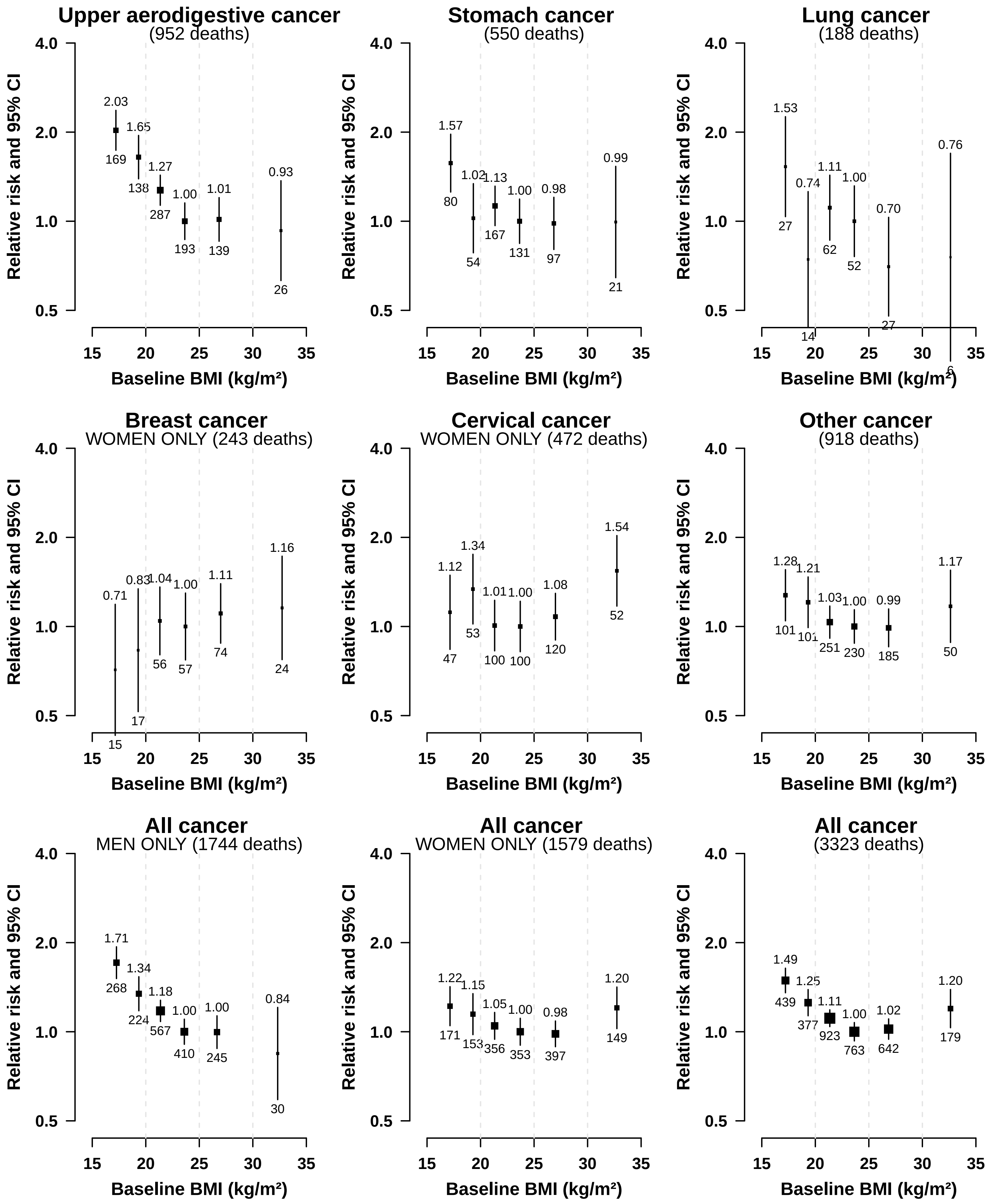


BMI=body−mass index. Death rate ratios (RR) at ages 35−69 years are adjusted for age, sex, education, socioeconomic status, smoking and alcohol. BMI categories: 15−18.5, 18.5−20, 20−22.5, 22.5−25 (reference), 25−30, 30−40 kg/m². For each category, area of the square is inversely proportional to the variance of the category−specific log risk, which also determines the confidence interval (CI). RR shown above each square and the number of events below. Exclusions as in Table S2, with further exclusion of the first 2 years of follow−up.

## Figure S6: Cause−specific mortality by SBP at ages 35−69
## EXCLUDING PRIOR DIABETES

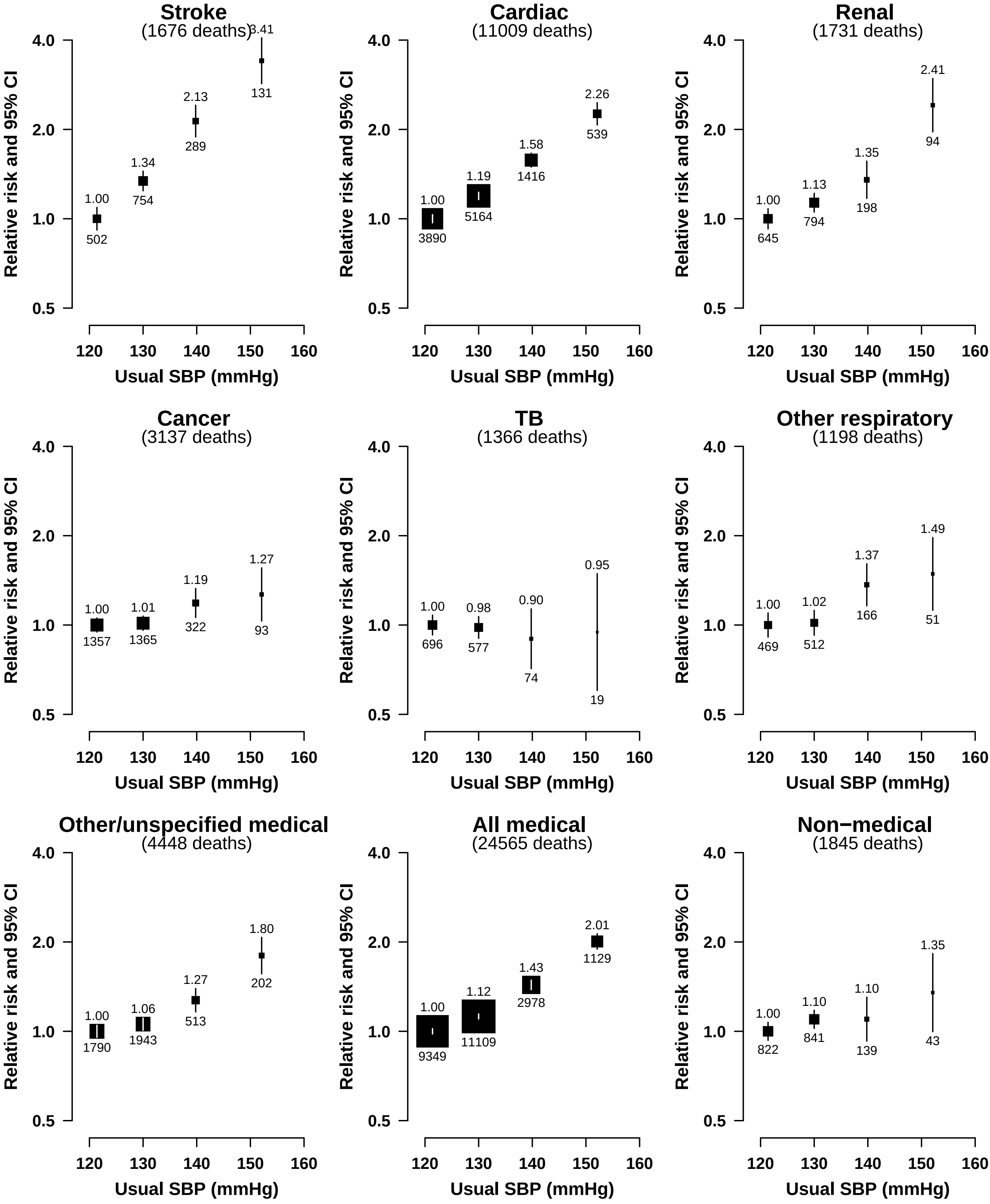


SBP=systolic blood pressure. Death rate ratios (RR) at ages 35−69 years are adjusted for age, sex, education, socioeconomic status, smoking, alcohol and BMI. For each category, area of the square is inversely proportional to the variance of the category−specific log risk, which also determines the confidence interval (CI). RR shown above each square and the number of events below. Exclusions as in Table S2, with further exclusion of prior diabetes and the first 2 years of follow−up.

# Figure S7: Cause−specific mortality by SBP at ages 35−69
# EXCLUDING SMOKERS, DRINKERS AND CHEWERS

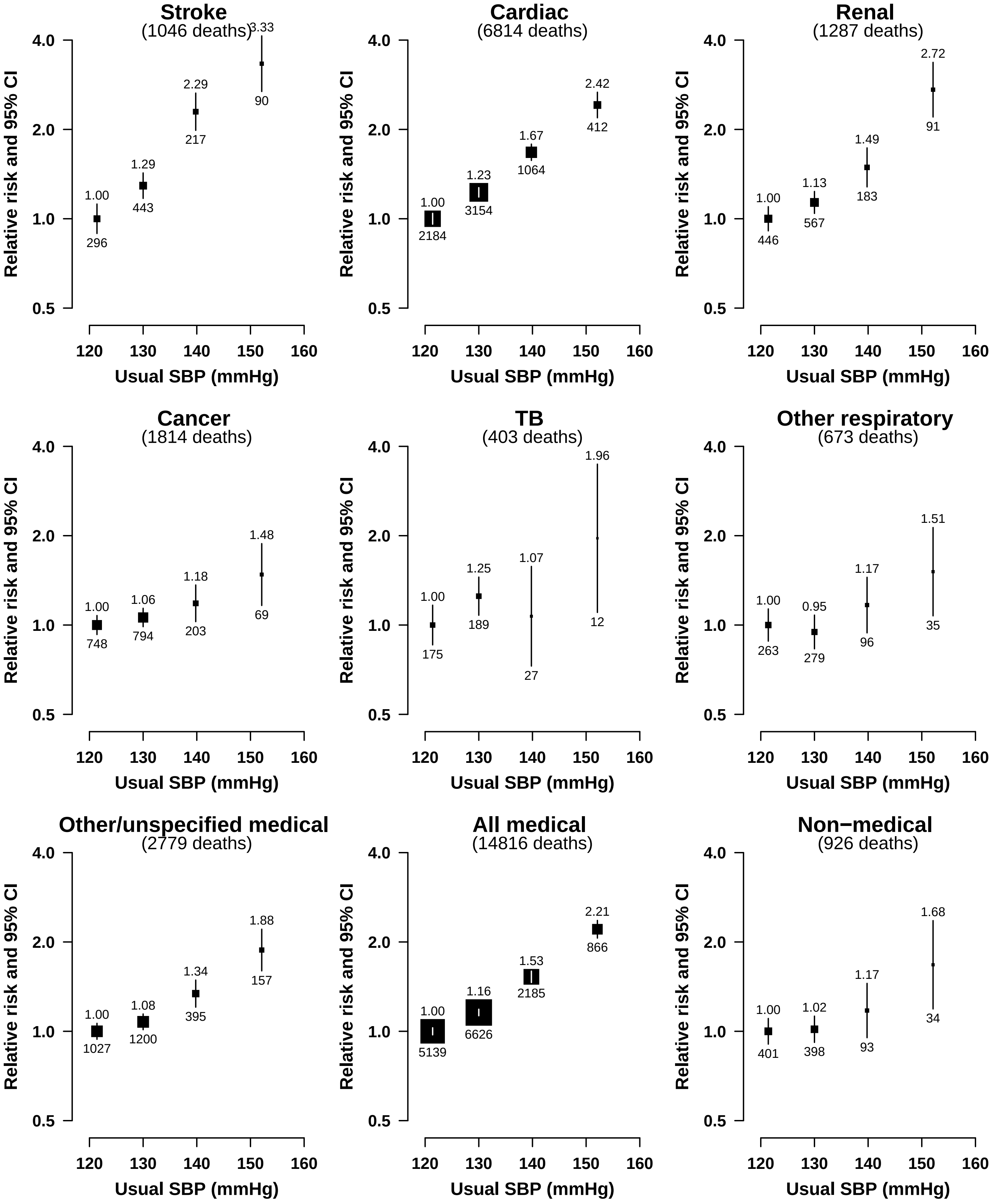


SBP=systolic blood pressure. Death rate ratios (RR) at ages 35−69 years are adjusted for age, sex, education, socioeconomic status and BMI. For each category, area of the square is inversely proportional to the variance of the category−specific log risk, which also determines the confidence interval (CI). RR shown above each square and the number of events below. Exclusions as in Table S2, with further exclusion of ever smokers, quid chewers, ever−weekly drinkers, and the first 2 years of follow−up.

# Figure S8: Cause−specific mortality by SBP at ages 35−69
# EXCLUDING FIRST 5 YEARS OF FOLLOW−UP

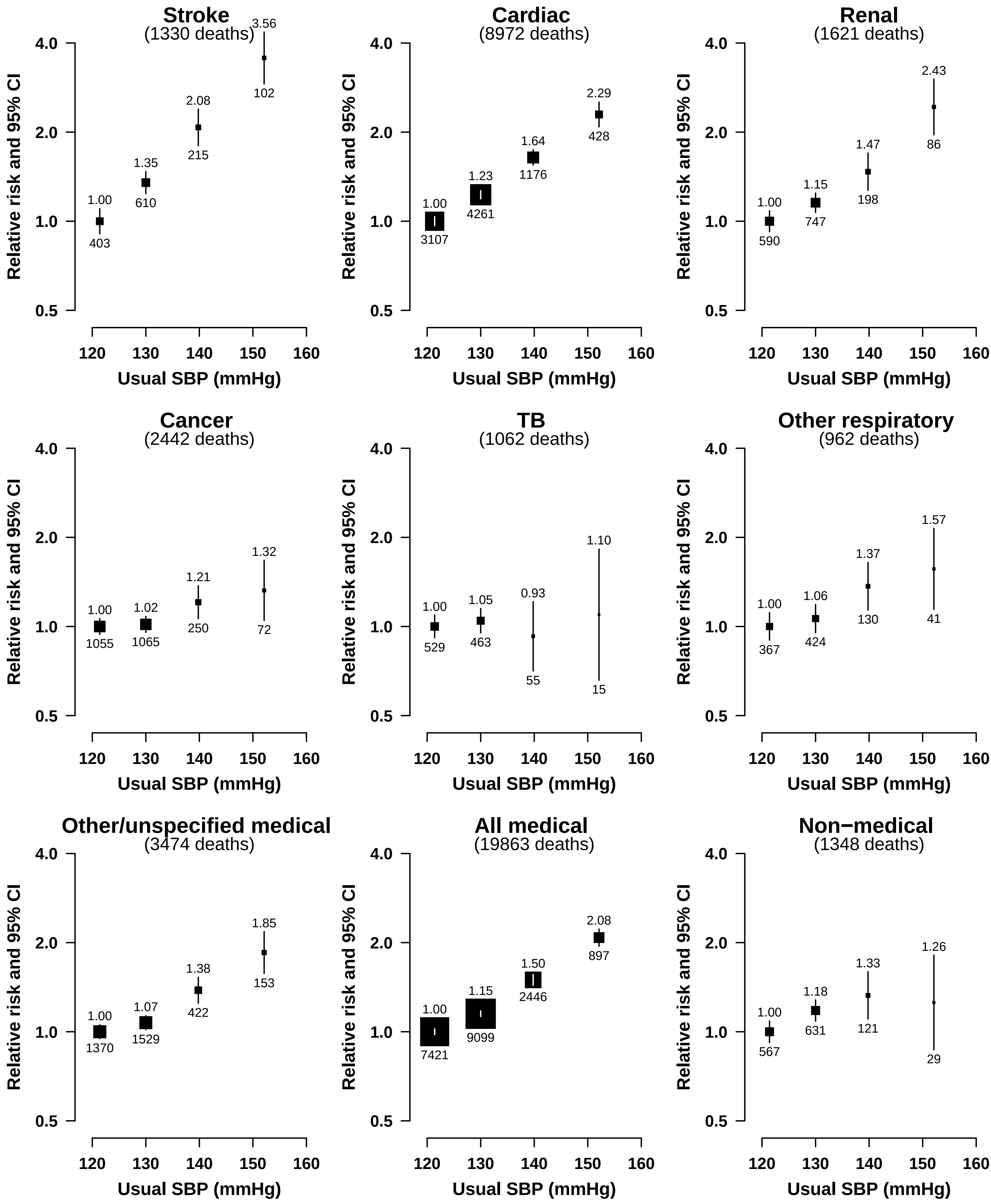


SBP=systolic blood pressure. Death rate ratios (RR) at ages 35−69 years are adjusted for age, sex, education, socioeconomic status, smoking, alcohol and BMI. For each category, area of the square is inversely proportional to the variance of the category−specific log risk, which also determines the confidence interval (CI). RR shown above each square and the number of events below. Exclusions as in Table S2, with further exclusion of the first 5 years of follow−up.

## Figure S9: Cause−specific mortality by SBP at ages 35−69
## MEN ONLY

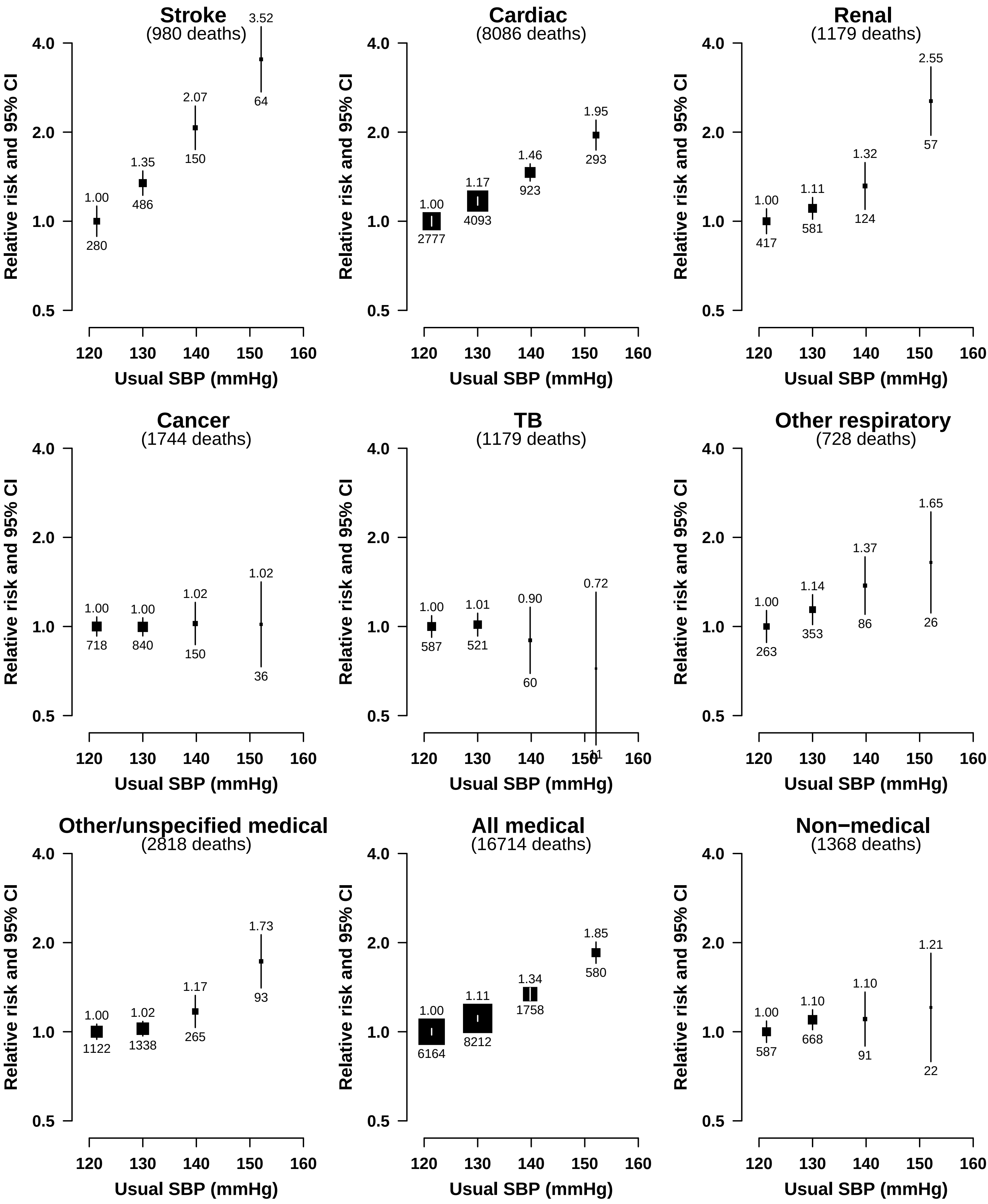


SBP=systolic blood pressure. Death rate ratios (RR) at ages 35−69 years are adjusted for age, education, socioeconomic status, smoking, alcohol and BMI. For each category, area of the square is inversely proportional to the variance of the category−specific log risk, which also determines the confidence interval (CI). RR shown above each square and the number of events below. Exclusions as in Table S2, with further exclusion of women and the first 2 years of follow−up.

## Figure S10: Cause−specific mortality by SBP at ages 35−69
## WOMEN ONLY

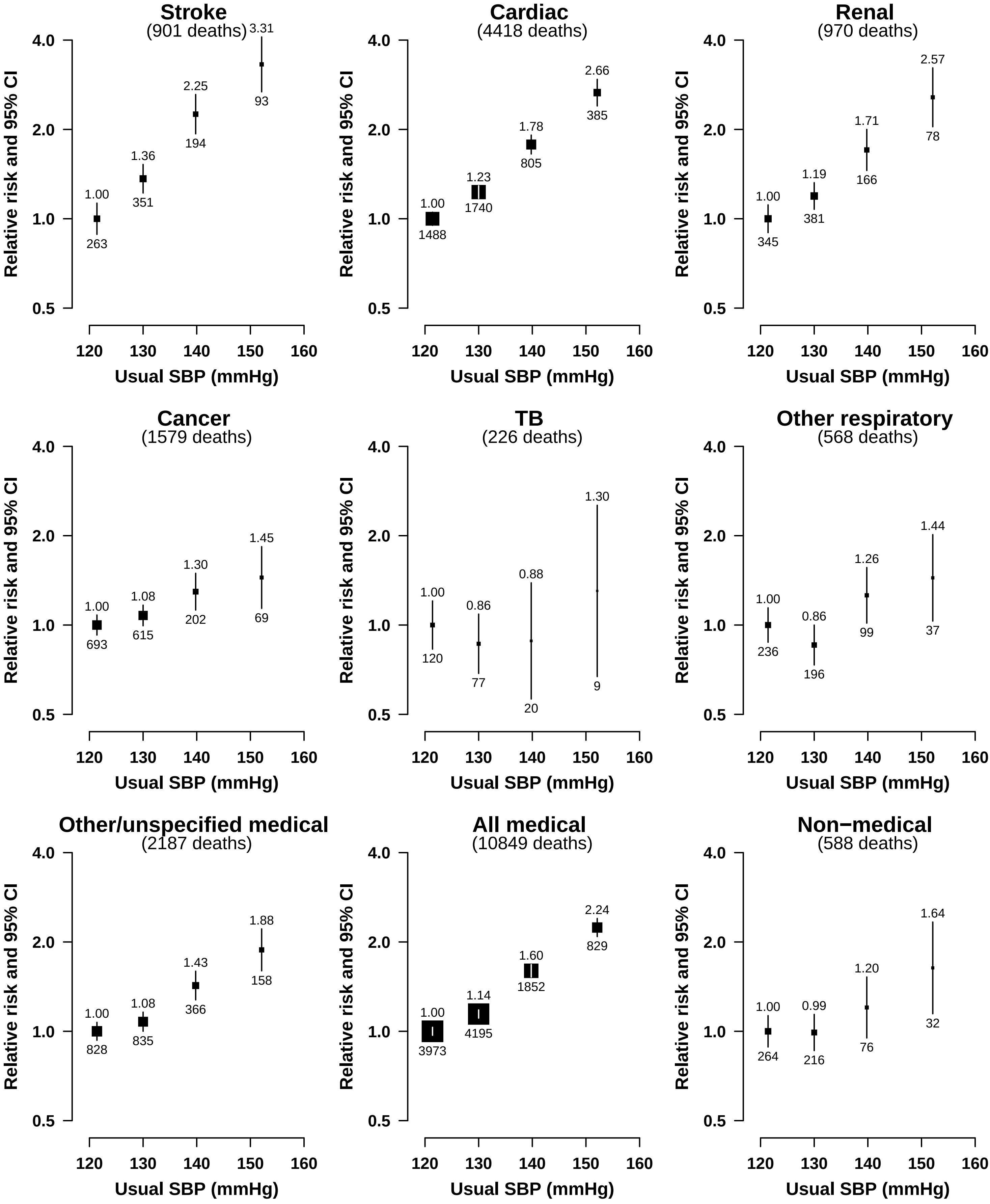


SBP=systolic blood pressure. Death rate ratios (RR) at ages 35−69 years are adjusted for age, education, socioeconomic status, smoking, alcohol and BMI. For each category, area of the square is inversely proportional to the variance of the category−specific log risk, which also determines the confidence interval (CI). RR shown above each square and the number of events below. Exclusions as in Table S2, with further exclusion of men and the first 2 years of follow−up.

## Figure S11: Cause−specific mortality by BMI at ages 35−69
## ADDITIONALLY ADJUSTED FOR QUID CHEWING

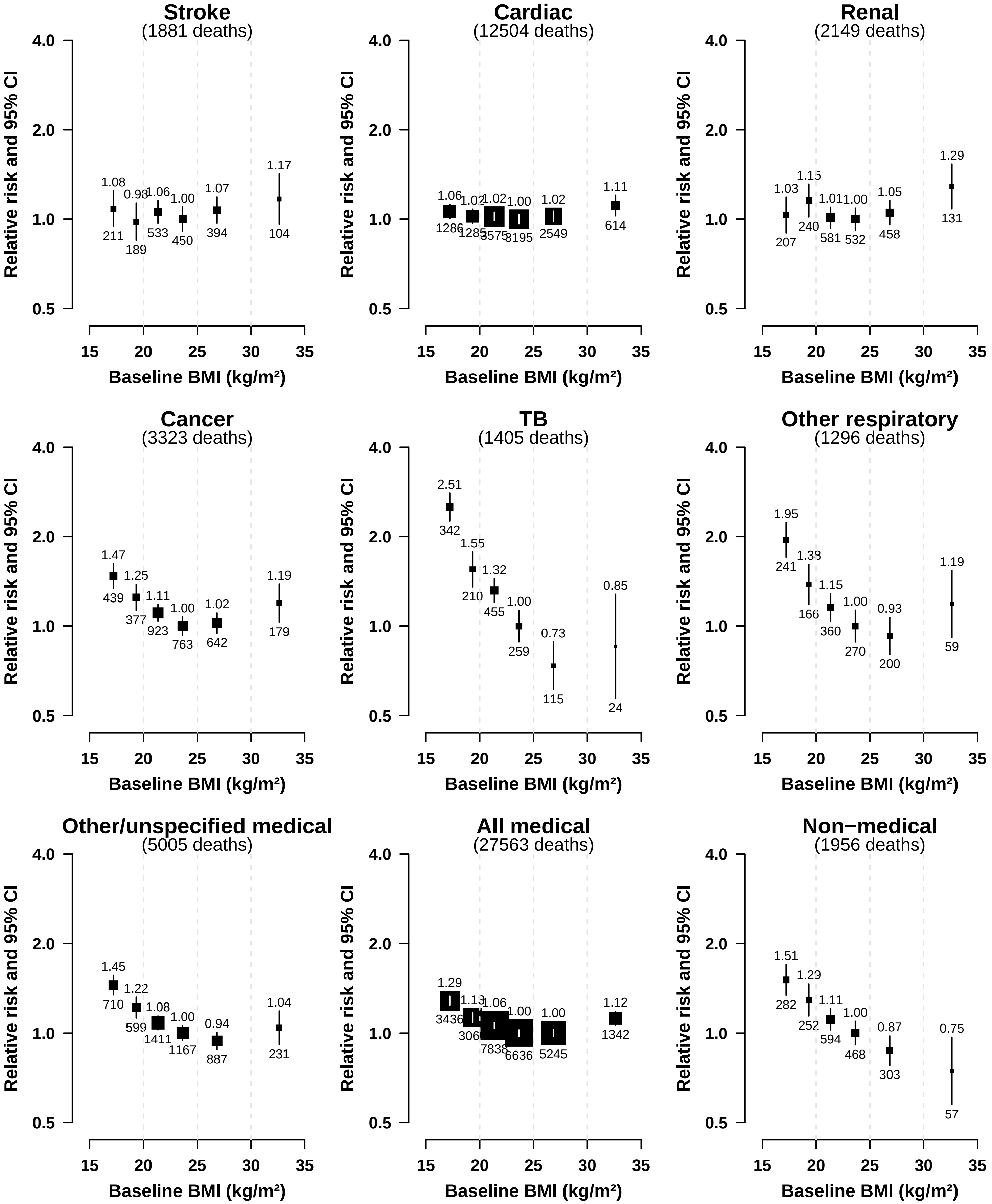


BMI=body−mass index. Death rate ratios (RR) at ages 35−69 years are adjusted for age, sex, education, socioeconomic status, smoking, alcohol and quid chewing. BMI categories: 15−18.5, 18.5−20, 20−22.5, 22.5−25 (reference), 25−30, 30−40 kg/m². For each category, area of the square is inversely proportional to the variance of the category−specific log risk, which also determines the confidence interval (CI). RR shown above each square and the number of events below. Exclusions as in Table S2, with further exclusion of the first 2 years of follow−up.

# Figure S12: Cause−specific mortality by BMI at ages 35−69
# EXCLUDING PRIOR DIABETES

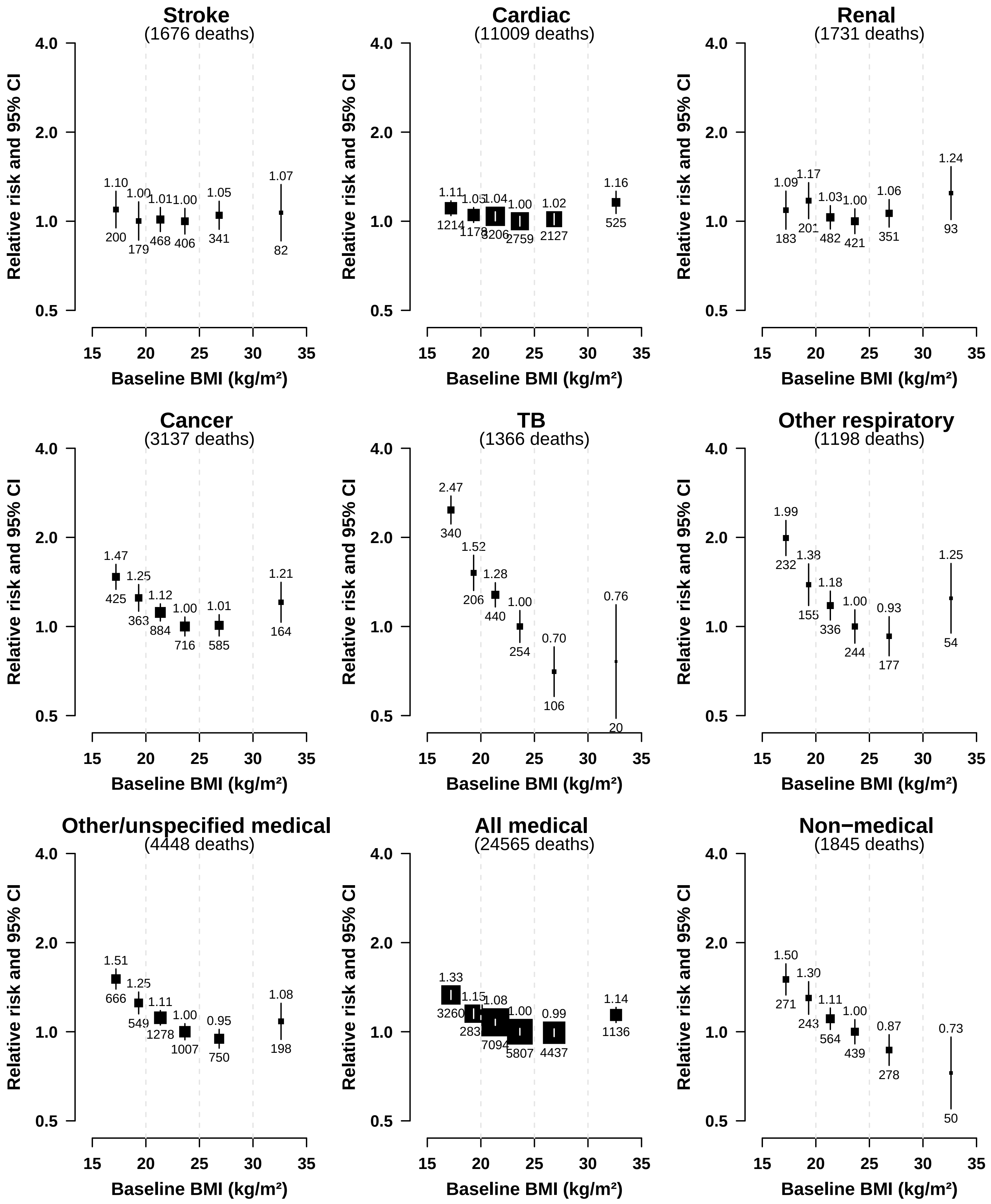


BMI=body−mass index. Death rate ratios (RR) at ages 35−69 years are adjusted for age, sex, education, socioeconomic status, smoking and alcohol. BMI categories: 15−18.5, 18.5−20, 20−22.5, 22.5−25 (reference), 25−30, 30−40 kg/m². For each category, area of the square is inversely proportional to the variance of the category−specific log risk, which also determines the confidence interval (CI). RR shown above each square and the number of events below. Exclusions as in Table S2, with further exclusion of prior diabetes and the first 2 years of follow−up.

# Figure S13: Cause−specific mortality by BMI at ages 35−69
# EXCLUDING EVER SMOKERS

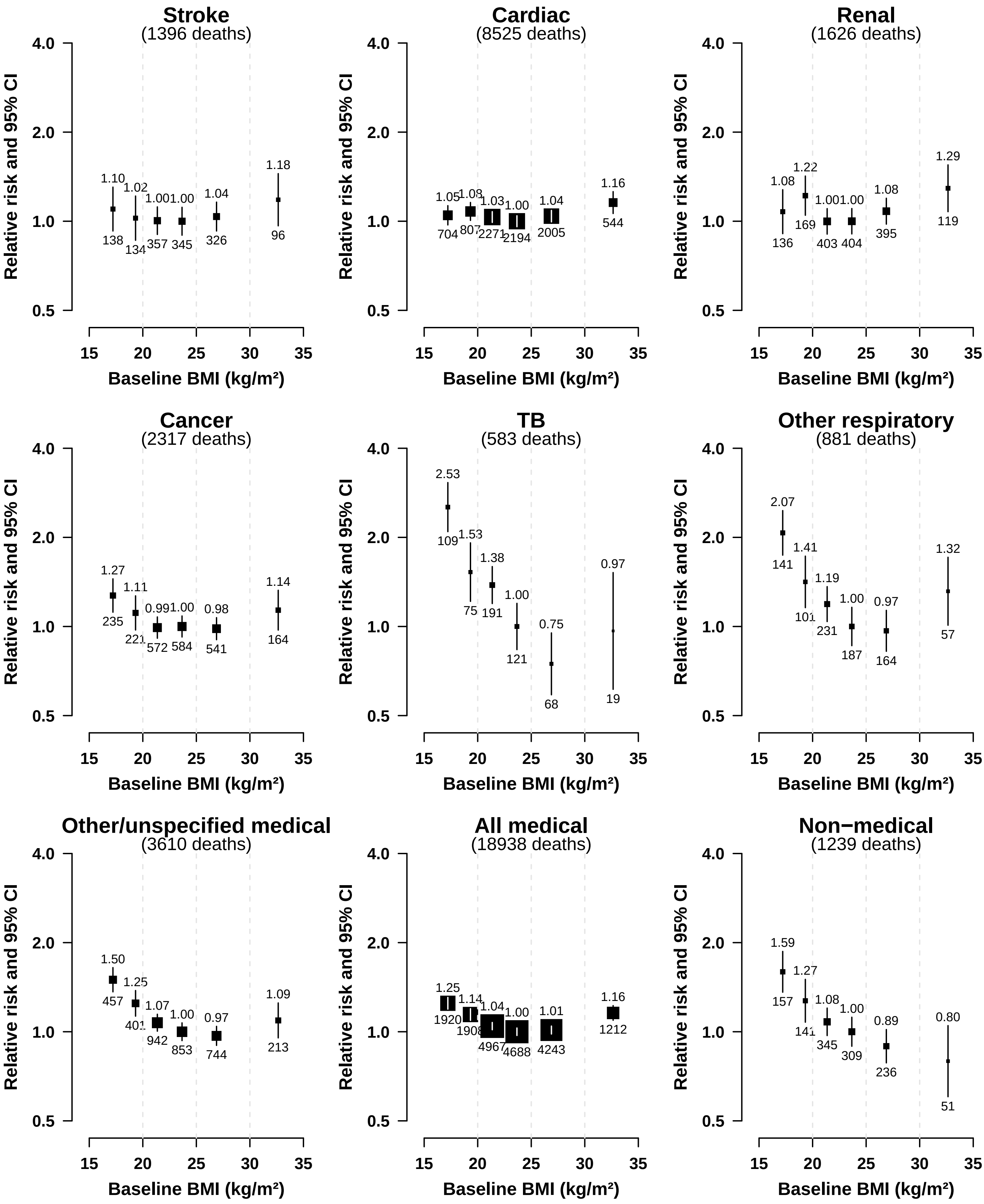


BMI=body−mass index. Death rate ratios (RR) at ages 35−69 years are adjusted for age, sex, education, socioeconomic status and alcohol. BMI categories: 15−18.5, 18.5−20, 20−22.5, 22.5−25 (reference), 25−30, 30−40 kg/m². For each category, area of the square is inversely proportional to the variance of the category−specific log risk, which also determines the confidence interval (CI). RR shown above each square and the number of events below. Exclusions as in Table S2, with further exclusion of ever smokers and the first 2 years of follow−up.

## Figure S14: Cause−specific mortality by BMI at ages 35−69
## EXCLUDING SMOKERS, DRINKERS AND QUID CHEWERS

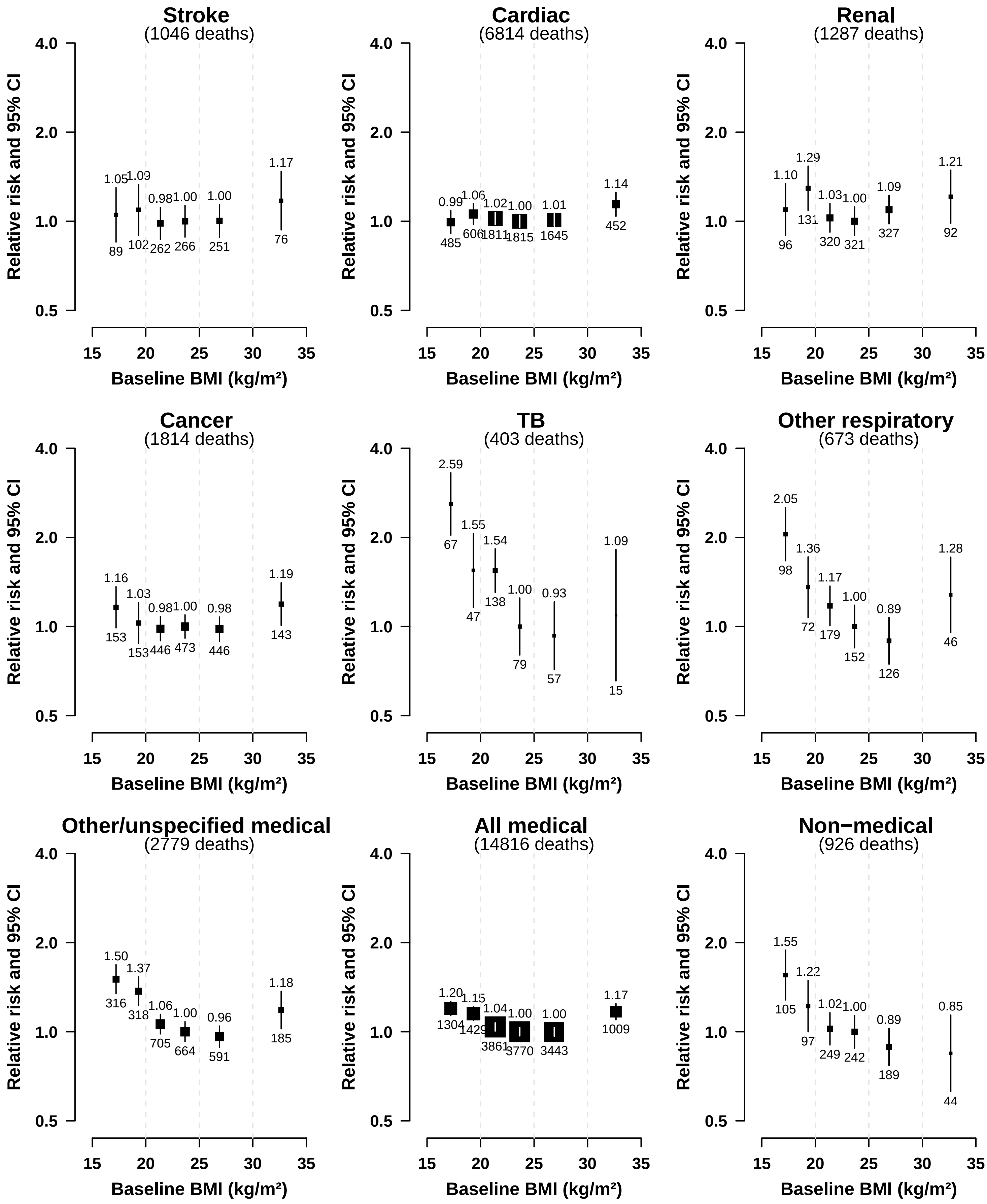


BMI=body−mass index. Death rate ratios (RR) at ages 35−69 years are adjusted for age, sex, education and socioeconomic status. BMI categories: 15−18.5, 18.5−20, 20−22.5, 22.5−25 (reference), 25−30, 30−40 kg/m². For each category, area of the square is inversely proportional to the variance of the category−specific log risk, which also determines the confidence interval (CI). RR shown above each square and the number of events below. Exclusions as in Table S2, with further exclusion of ever smokers, quid chewers, ever−weekly drinkers and the first 2 years of follow−up.

# Figure S15: Cause−specific mortality by BMI at ages 35−69
## EXCLUDING SMOKERS, DRINKERS AND CHEWERS
## PRIOR TO ADJUSTMENT FOR SOCIAL CLASS OR EDUCATION

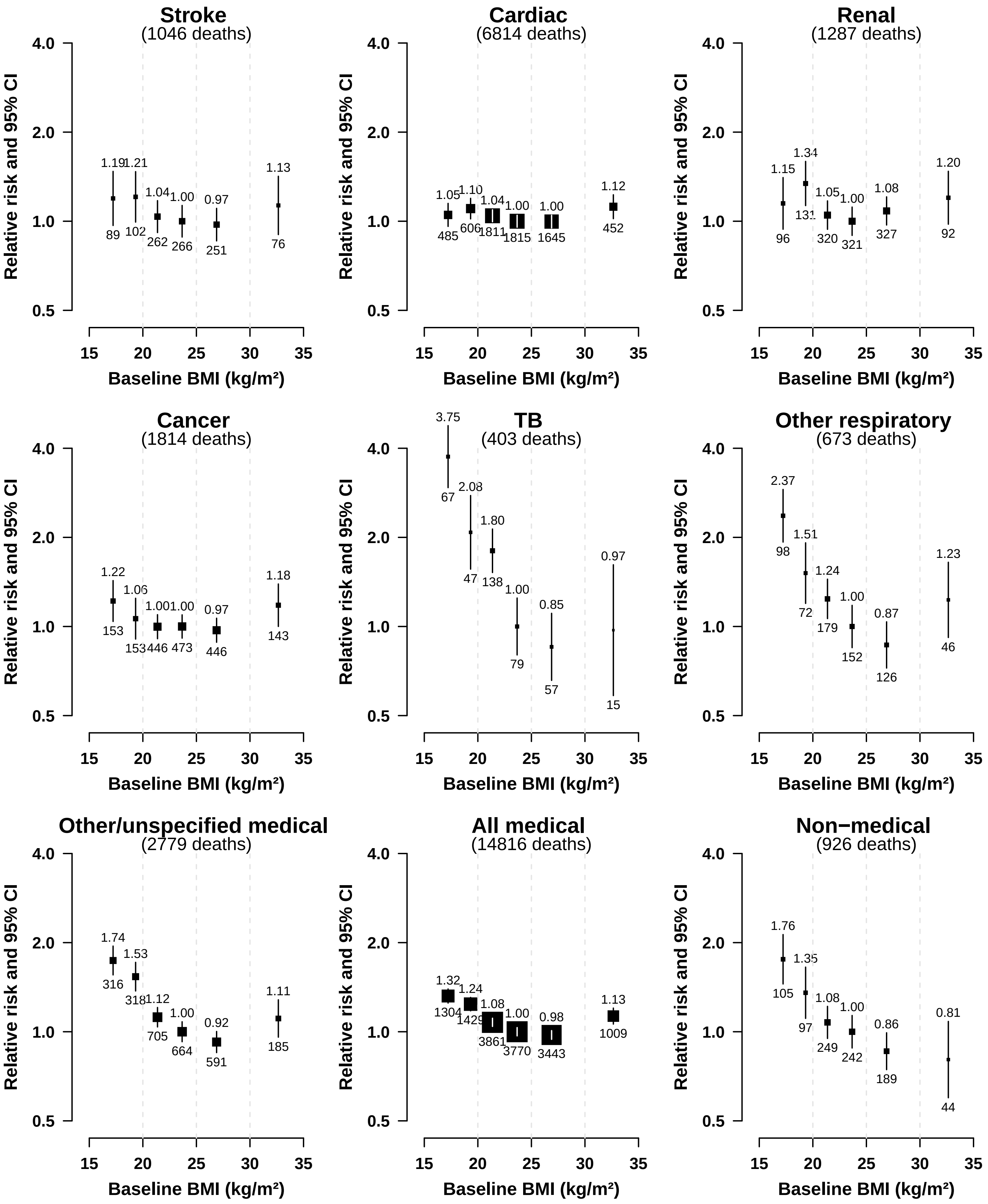


BMI=body−mass index, SBP=systolic blood pressure. Death rate ratios (RR) at ages 35−69 years are adjusted for age and sex. BMI categories: 15−18.5, 18.5−20, 20−22.5, 22.5−25 (reference), 25−30, 30−40 kg/m². For each category, area of the square is inversely proportional to the variance of the category−specific log risk, which also determines the confidence interval (CI). RR shown above each square and the number of events below. Exclusions as in Table S2, with additional exclusion for smoking, drinking and chewing.

# Figure S16: Cause−specific mortality by BMI at ages 35−69
## RESTRICTED TO THOSE OF LOWER SOCIOECONOMIC STATUS

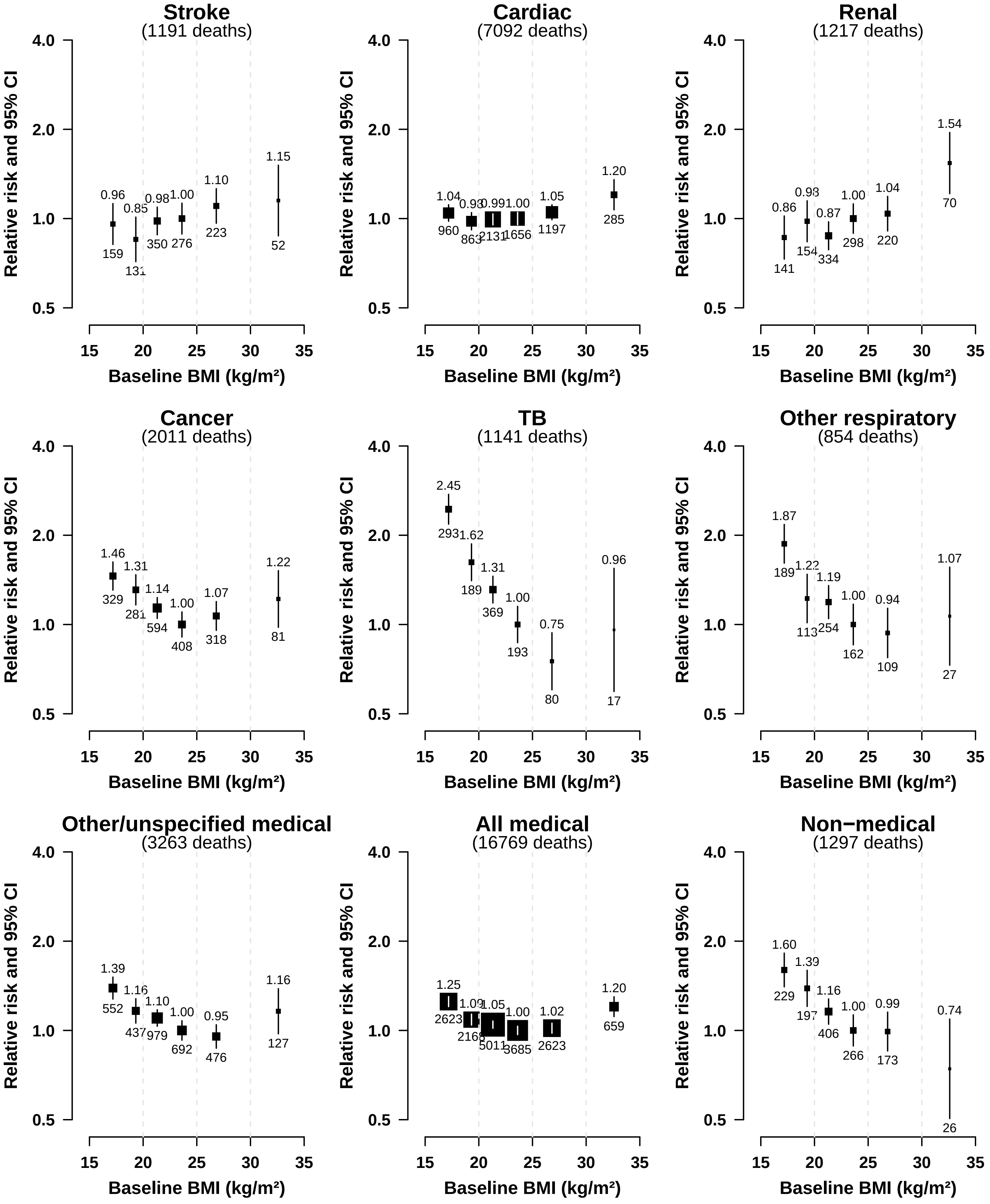


BMI=body−mass index. Death rate ratios (RR) at ages 35−69 years are adjusted for age, sex, education, socioeconomic status, smoking and alcohol. BMI categories: 15−18.5, 18.5−20, 20−22.5, 22.5−25 (reference), 25−30, 30−40 kg/m². For each category, area of the square is inversely proportional to the variance of the category−specific log risk, which also determines the confidence interval (CI). RR shown above each square and the number of events below. Exclusions as in Table s2, with further exclusion of the first 2 years of follow−up.

Lower socioeconomic status defined as in Table 1.

## Figure S17: Cause-specific mortality by BMI at ages 35-69
## RESTRICTED TO THOSE OF MEDIAN OR HIGHER SOCIOECONOMIC STATUS

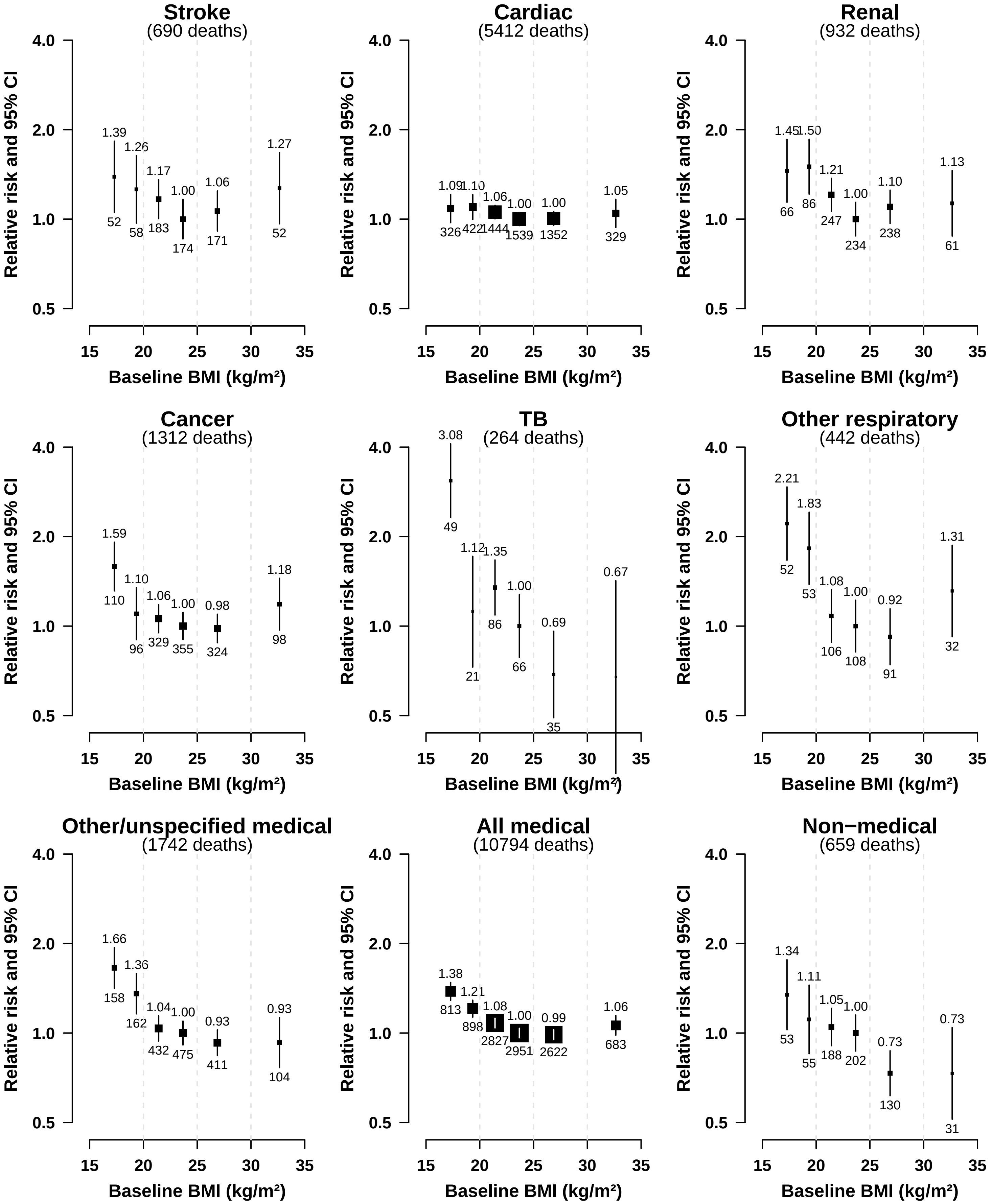


BMI=body−mass index. Death rate ratios (RR) at ages 35−69 years are adjusted for age, sex, education, socioeconomic status, smoking and alcohol. BMI categories: 15−18.5, 18.5−20, 20−22.5, 22.5−25 (reference), 25−30, 30−40 kg/m². For each category, area of the square is inversely proportional to the variance of the category−specific log risk, which also determines the confidence interval (CI). RR shown above each square and the number of events below. Exclusions as in Table s2, with further exclusion of the first 2 years of follow−up.

Median or higher socioeconomic status defined as in Table 1.

# Figure S18: Cause−specific mortality by BMI at ages 35−69
# EXCLUDING FIRST 5 YEARS FOLLOW−UP

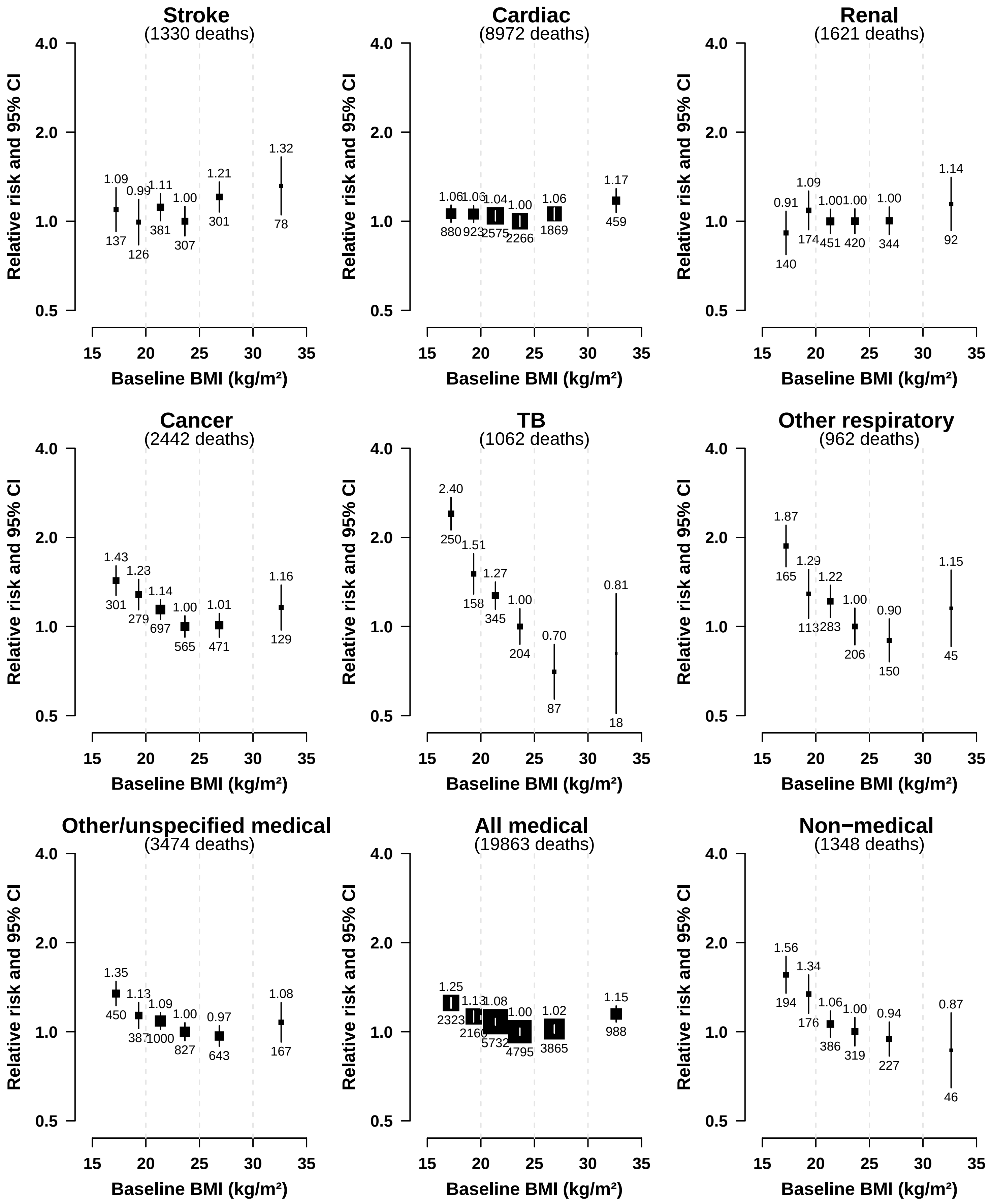


BMI=body−mass index. Death rate ratios (RR) at ages 35−69 years are adjusted for age, sex, education, socioeconomic status, smoking and alcohol. BMI categories: 15−18.5, 18.5−20, 20−22.5, 22.5−25 (reference), 25−30, 30−40 kg/m². For each category, area of the square is inversely proportional to the variance of the category−specific log risk, which also determines the confidence interval (CI). RR shown above each square and the number of events below. Exclusions as in Table S2, with further exclusion of the first 5 years of follow−up.

# Figure S19: Cause−specific mortality by BMI at ages 35−69
## MEN ONLY

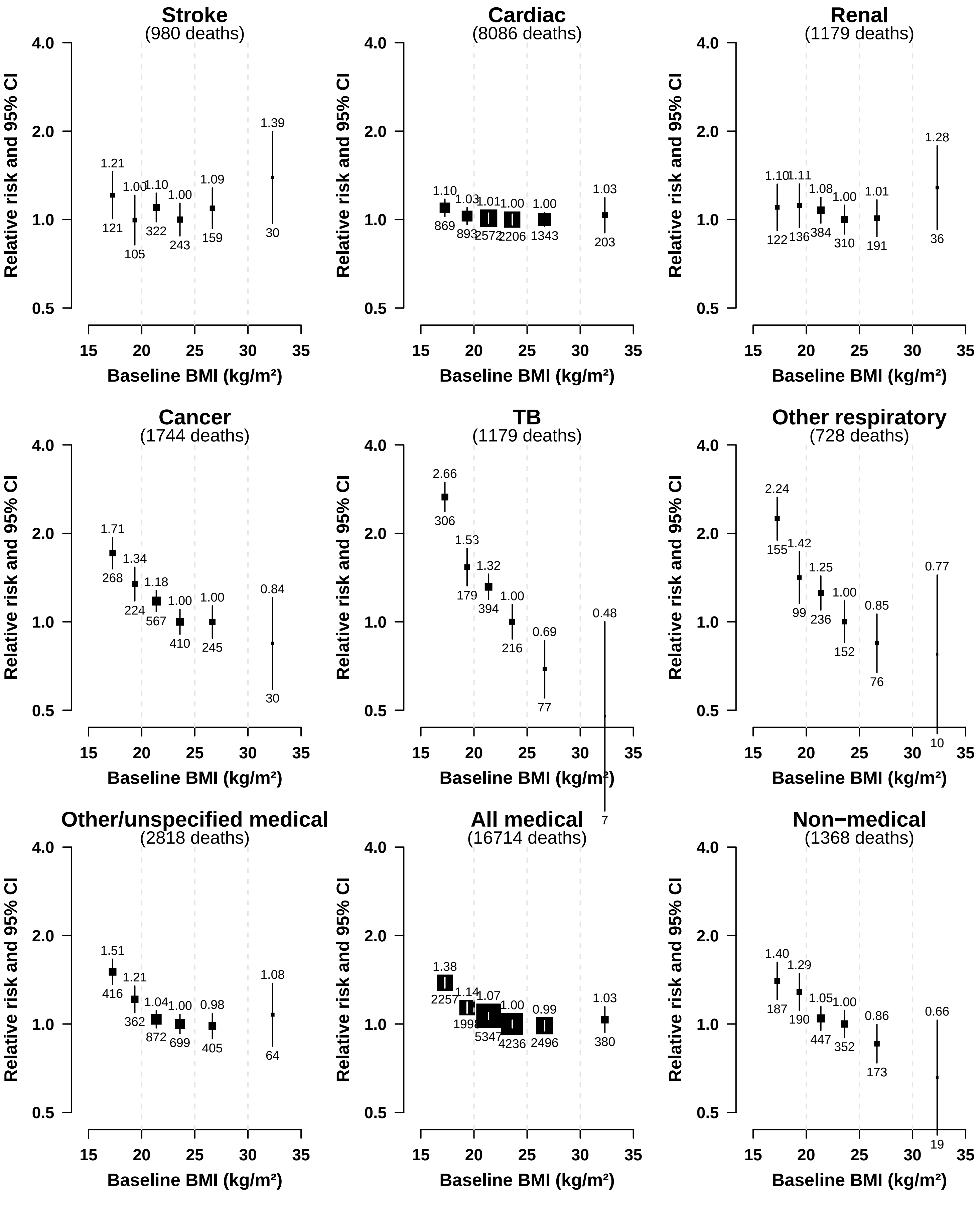


BMI=body−mass index. Death rate ratios (RR) at ages 35−69 years are adjusted for age, education, socioeconomic status, smoking and alcohol. BMI categories: 15−18.5, 18.5−20, 20−22.5, 22.5−25 (reference), 25−30, 30−40 kg/m². For each category, area of the square is inversely proportional to the variance of the category−specific log risk, which also determines the confidence interval (CI). RR shown above each square and the number of events below. Exclusions as in Table S2, with further exclusion of women and the first 2 years of follow−up.

# Figure S20: Cause−specific mortality by BMI at ages 35−69
## WOMEN ONLY

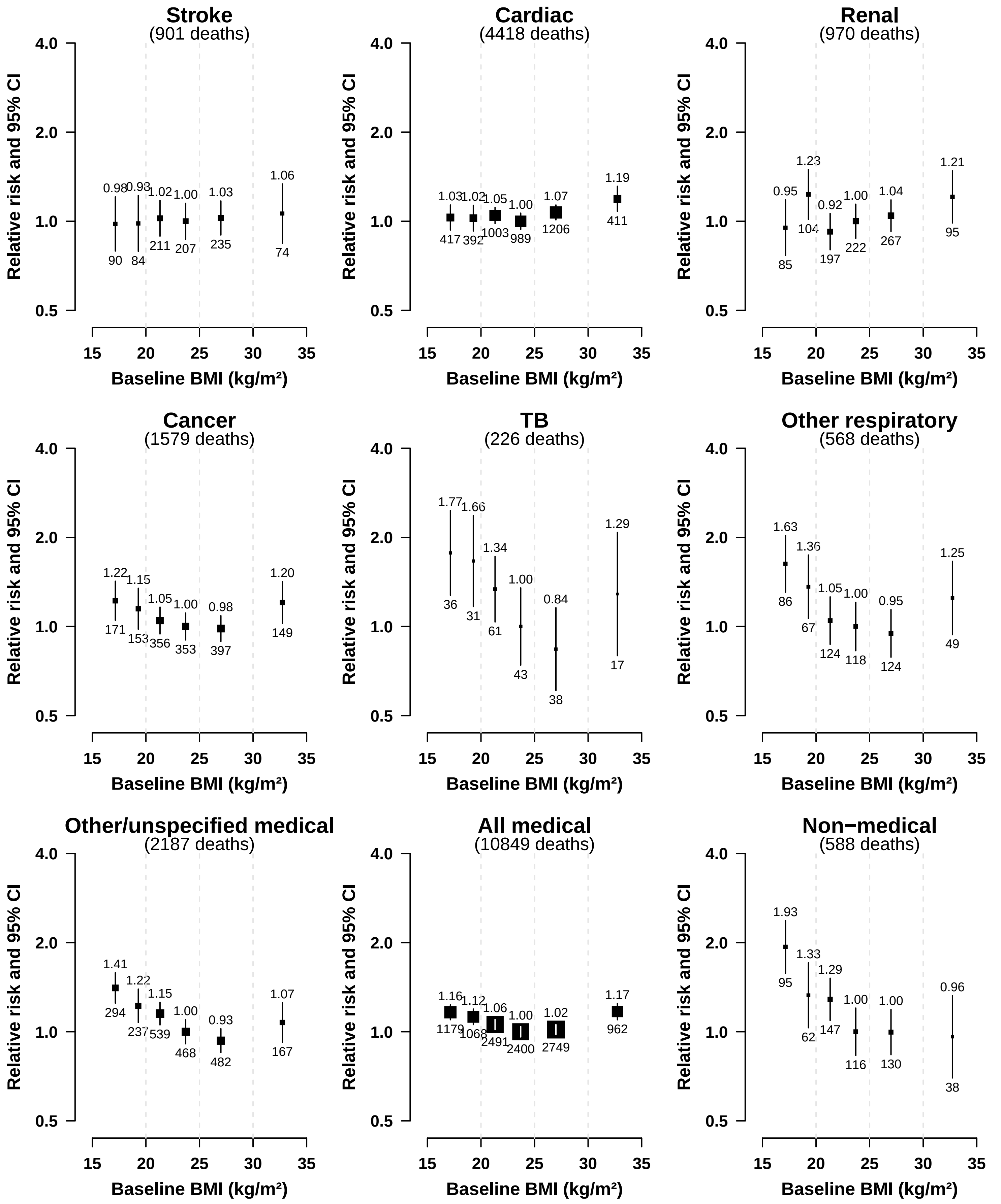


BMI=body−mass index. Death rate ratios (RR) at ages 35−69 years are adjusted for age, education, socioeconomic status, smoking and alcohol. BMI categories: 15−18.5, 18.5−20, 20−22.5, 22.5−25 (reference), 25−30, 30−40 kg/m². For each category, area of the square is inversely proportional to the variance of the category−specific log risk, which also determines the confidence interval (CI). RR shown above each square and the number of events below. Exclusions as in Table S2, with further exclusion of men and the first 2 years of follow−up.

# Figure S21: All vascular and all−cause mortality by waist circumference at ages 35−69 years (men only), before and after adjustment for usual SBP

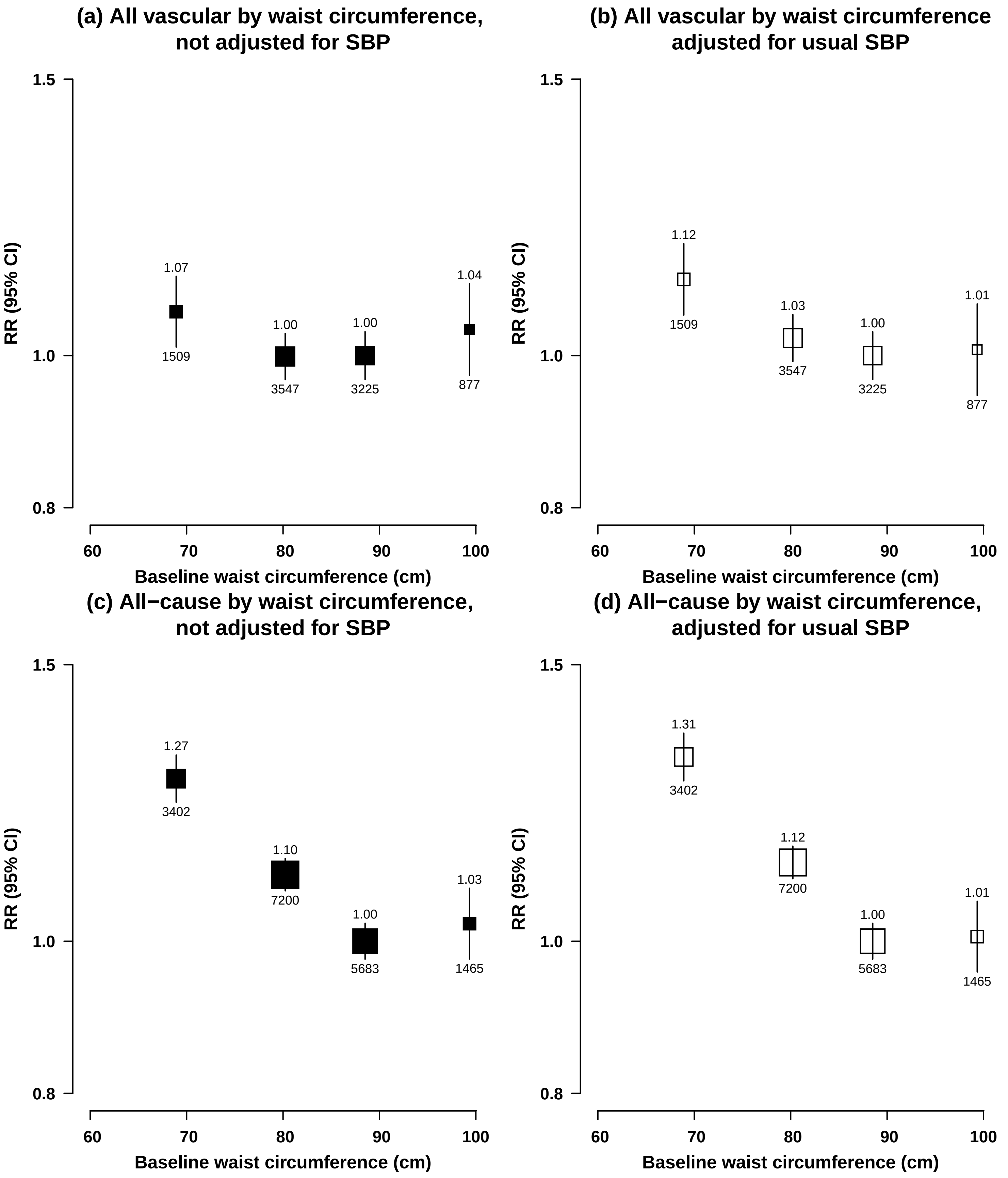


SBP=systolic blood pressure. Death rate ratios (RR) at ages 35−69 years are adjusted for age, education, socioeconomic status, smoking and alcohol, with further adjustment for usual SBP where indicated. Waist circumference categories: <75, 75−85, 85−95 (reference), 95+ cm. For each category, area of the square is inversely proportional to the variance of the category−specific log risk, which also determines the confidence interval (CI). RR shown above each square and the number of events below. Exclusions as in Table S2, with further exclusion of the first 2 years of follow−up.

## Figure S22: Cause−specific mortality by DBP at ages 35−69

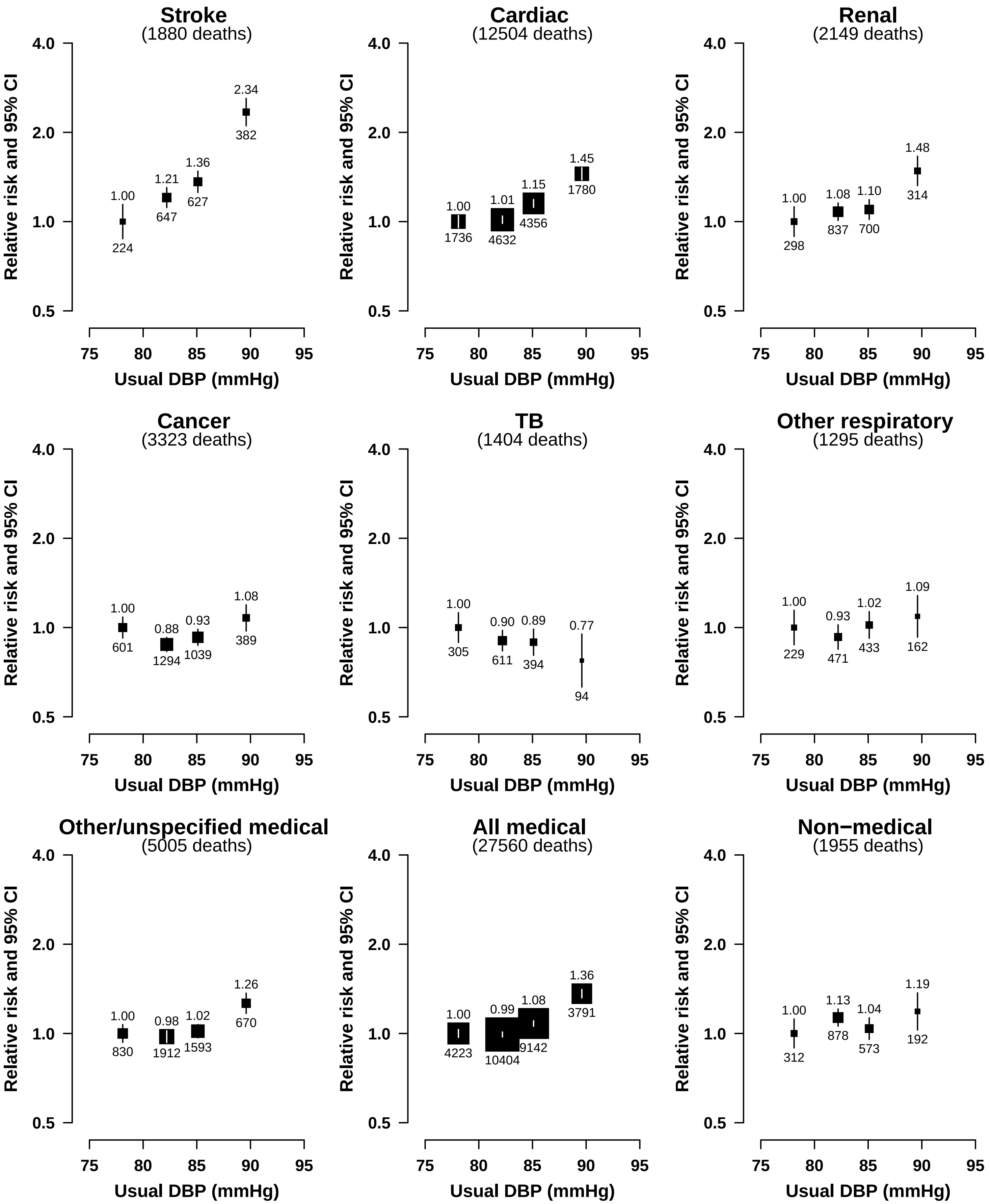


DBP=diastolic blood pressure. Death rate ratios (RR) at ages 35−69 years are adjusted for age, sex, education, socioeconomic status, smoking, alcohol and BMI. For each category, area of the square is inversely proportional to the variance of the category−specific log risk, which also determines the confidence interval (CI). RR shown above each square and the number of events below. Exclusions as in Table S2, with further exclusion of the first 2 years of follow−up.

## Figure S23: Cause−specific mortality by DBP at ages 70−79

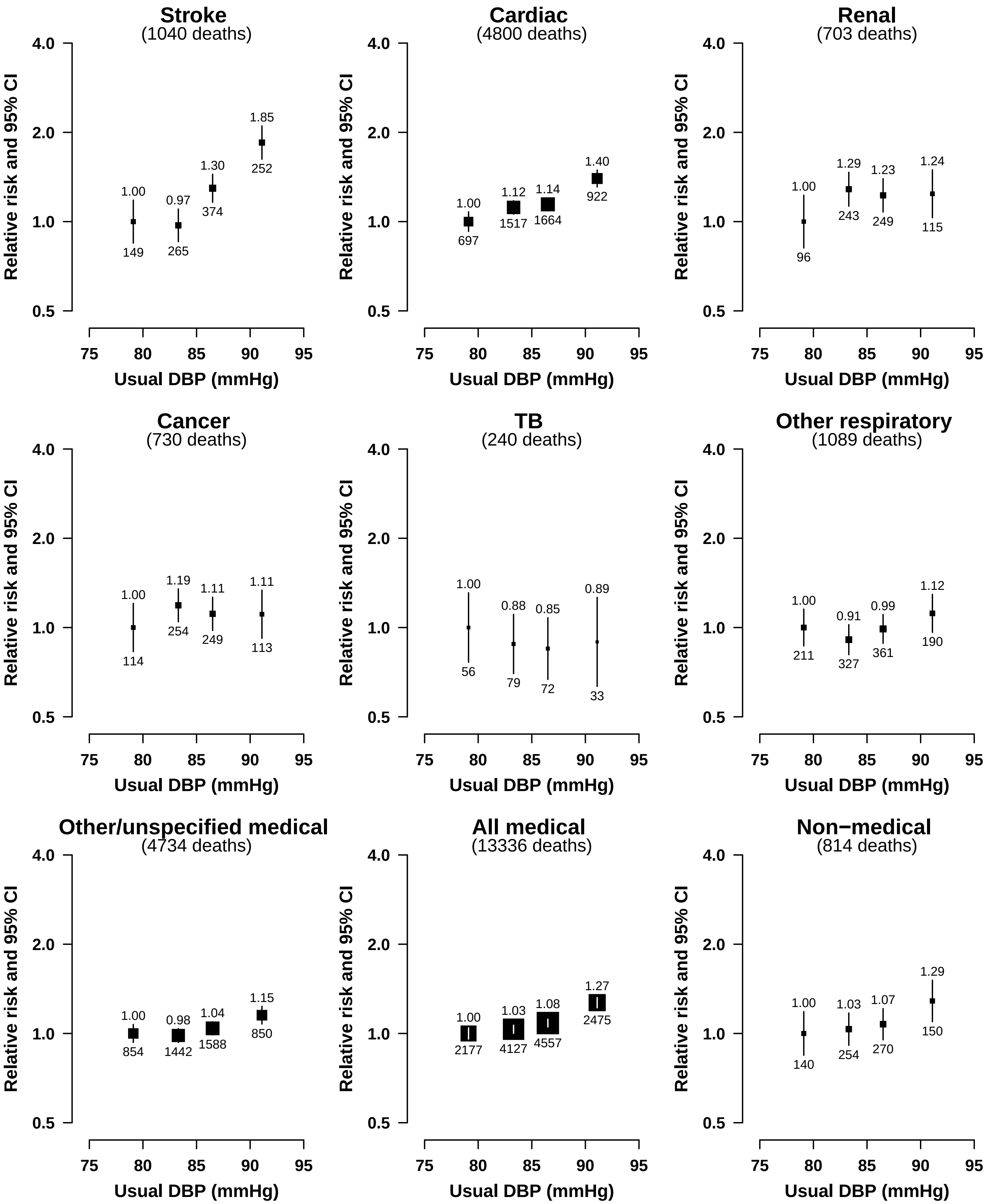


DBP=diastolic blood pressure. Death rate ratios (RR) at ages 70−79 years are adjusted for age, sex, education, socioeconomic status, smoking, alcohol and BMI. For each category, area of the square is inversely proportional to the variance of the category−specific log risk, which also determines the confidence interval (CI). RR shown above each square and the number of events below. Exclusions as in Table S2, with further exclusion of the first 2 years of follow−up.

# Figure S24: Cause−specific mortality by SBP at ages 70−79

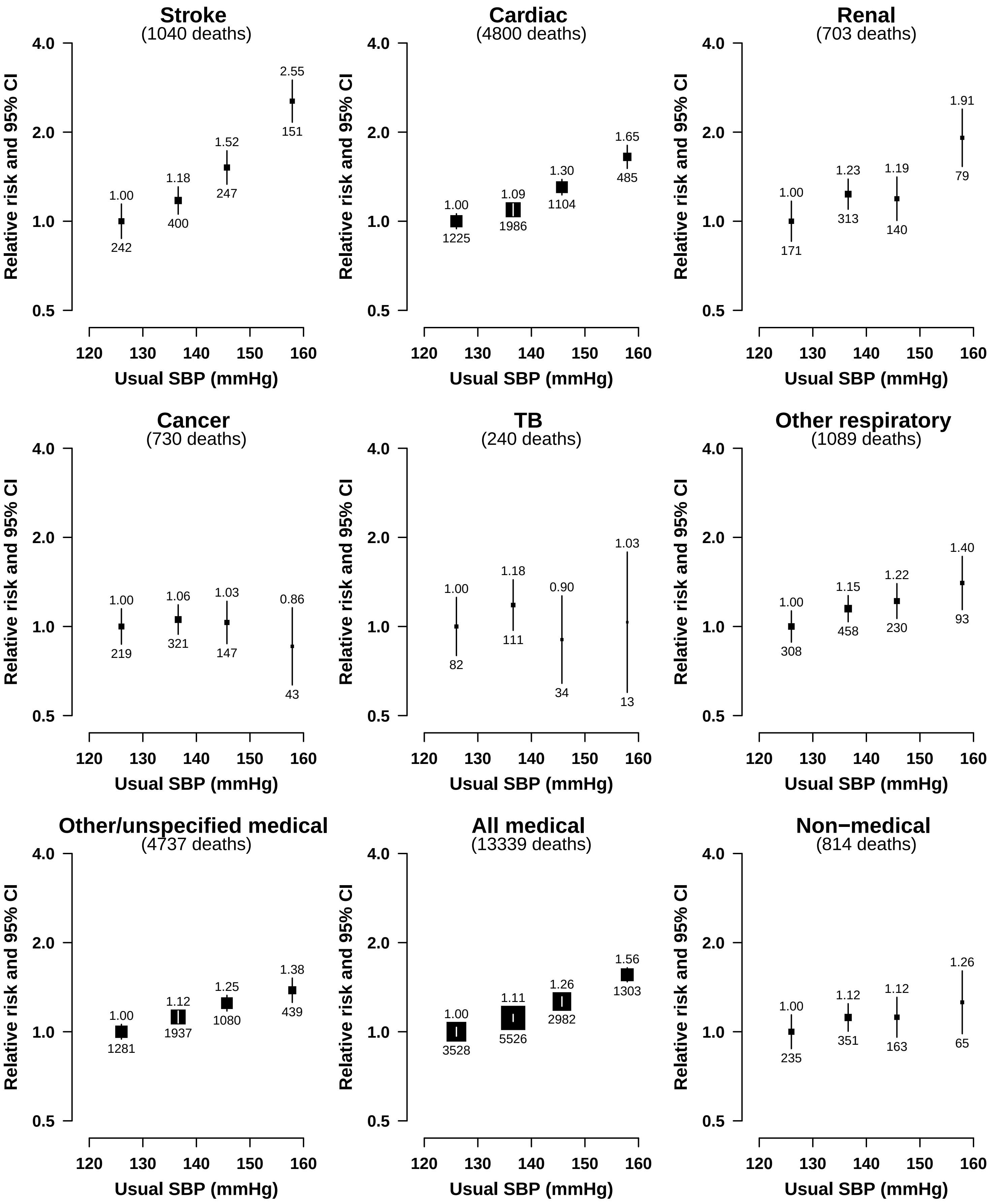


SBP=systolic blood pressure. Death rate ratios (RR) at ages 70−79 years are adjusted for age, sex, education, socioeconomic status, smoking, alcohol and BMI. For each category, area of the square is inversely proportional to the variance of the category−specific log risk, which also determines the confidence interval (CI). RR shown above each square and the number of events below. Exclusions as in Table S2, except that this is restricted to those with follow−up at ages 70−79 rather than 35−69, with further exclusion of the first 2 years of follow−up.

# Figure S25: Cause−specific mortality by BMI at ages 70−79

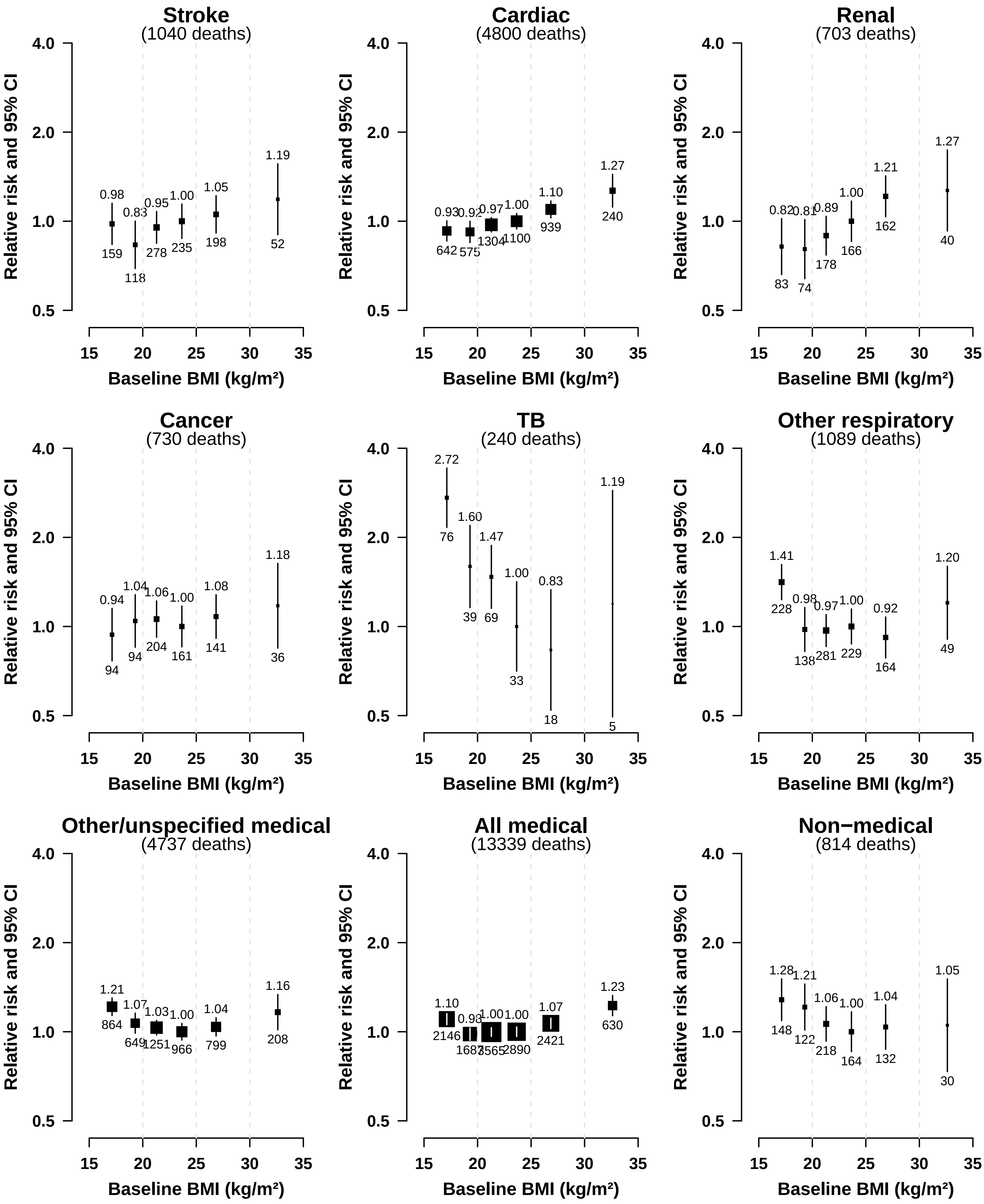


BMI=body−mass index. Death rate ratios (RR) at ages 70−79 years are adjusted for age, sex, education, socioeconomic status, smoking and alcohol. BMI categories: 15−18.5, 18.5−20, 20−22.5, 22.5−25 (reference), 25−30, 30−40 kg/m². For each category, area of the square is inversely proportional to the variance of the category−specific log risk, which also determines the confidence interval (CI). RR shown above each square and the number of events below. Exclusions as in Table S2, except that this is restricted to those with follow−up at ages 70−79 rather than 35−69, with further exclusion of the first 2 years of follow−up.

## Figure S26: Cause−specific mortality by BMI at ages 70−79 additionally adjusted for usual SBP

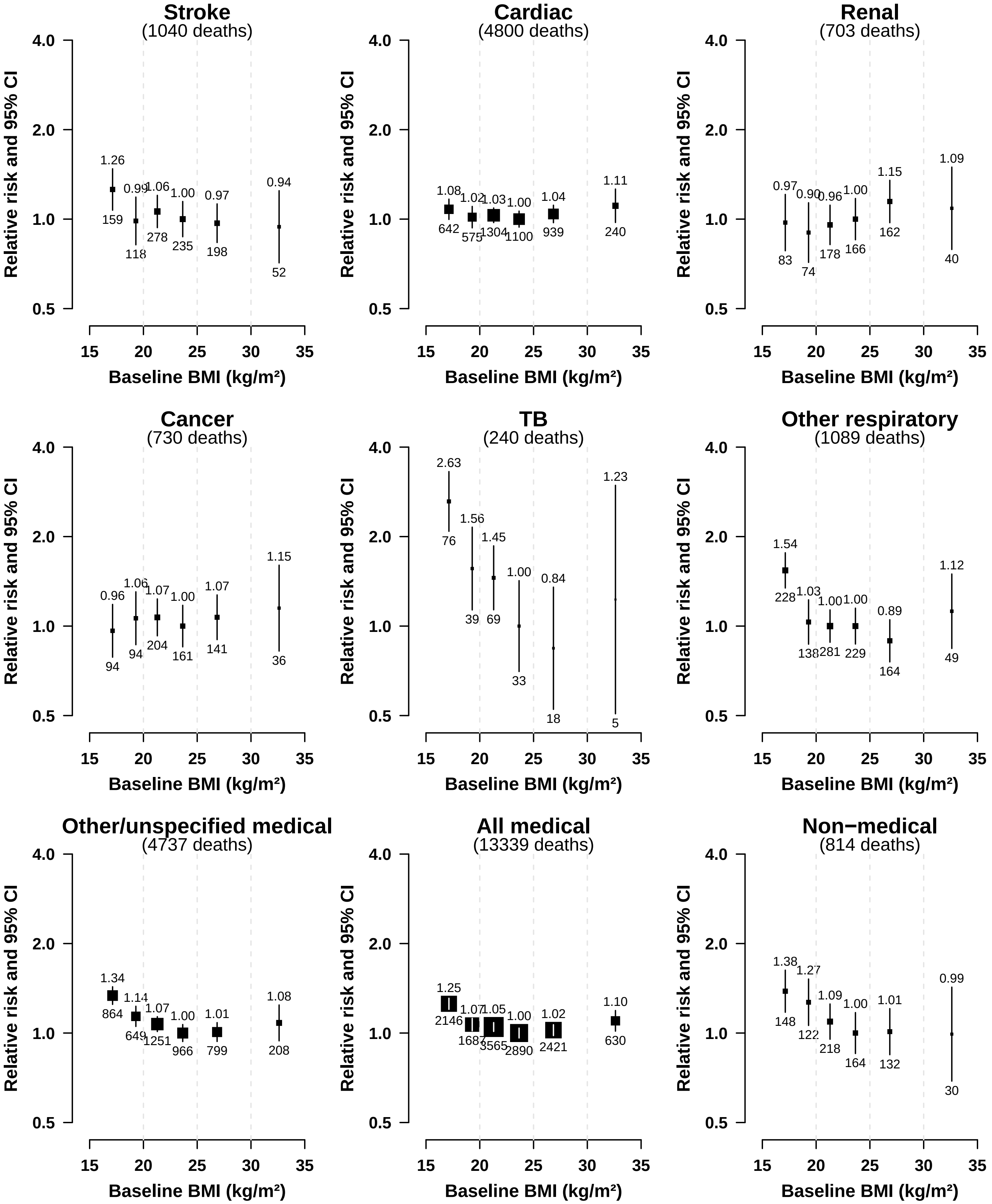


BMI=body−mass index. Death rate ratios (RR) at ages 70−79 years are adjusted for age, sex, education, socioeconomic status, smoking and alcohol. BMI categories: 15−18.5, 18.5−20, 20−22.5, 22.5−25 (reference), 25−30, 30−40 kg/m². For each category, area of the square is inversely proportional to the variance of the category−specific log risk, which also determines the confidence interval (CI). RR shown above each square and the number of events below. Exclusions as in Table S2, with further exclusion of the first 2 years of follow−up. ADDITIONALLY ADJUSTED FOR USUAL SBP

# Figure S27: BMI vs stroke mortality rate in Chennai and in Europe/N. America (excluding smokers)

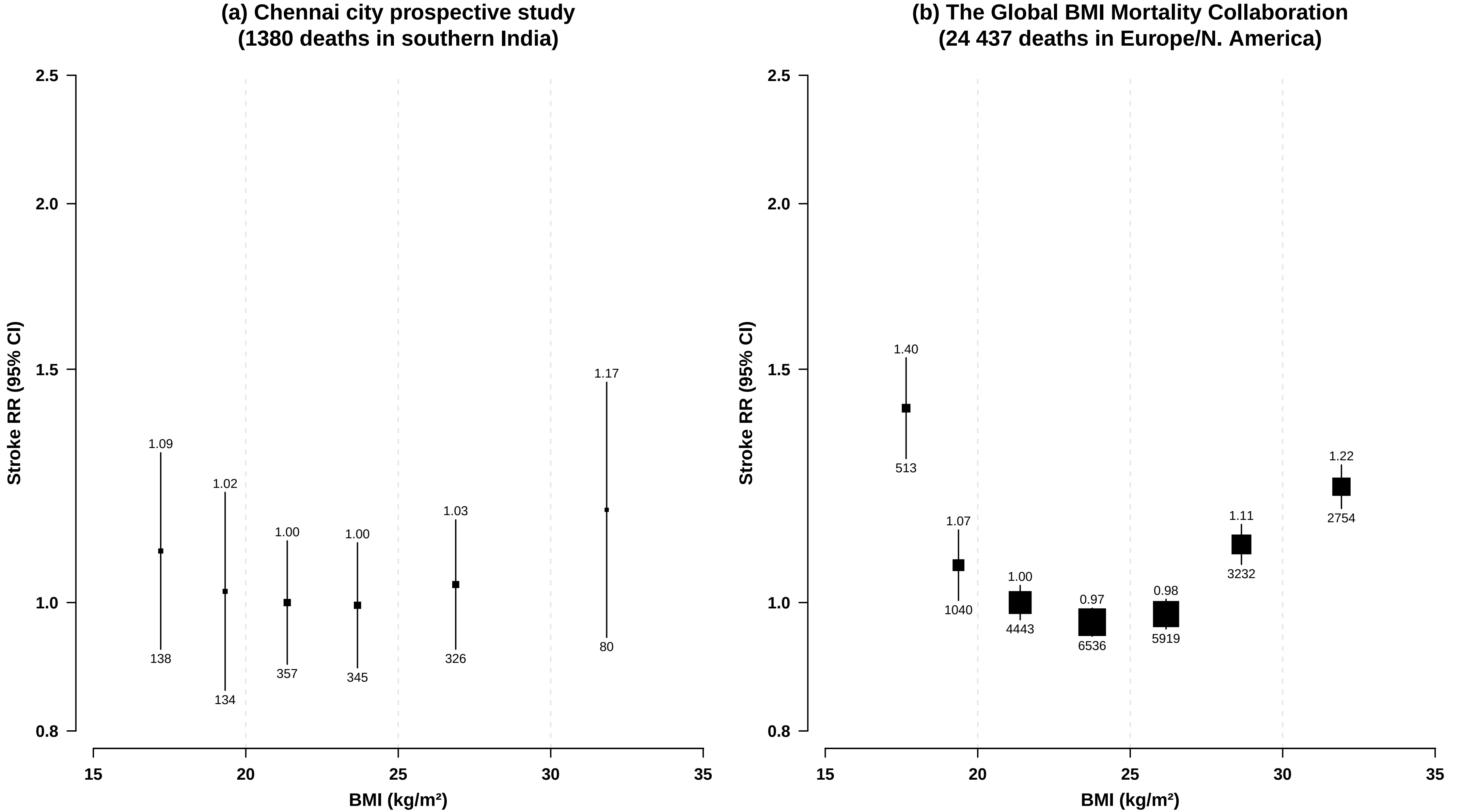


RR: Rate ratio for death from any cause, adjusted for major confounders, excluding early follow-up (2 years in Chennai, 5 years in N America/Europe) and those with prior chronic disease at baseline. Analyses exclude BMI<15 and BMI>35. Estimates for Europe/N America are from the Global BMI Mortality Collaboration. Conventions as in Figure 1.tes for Europe/N America are from the Global BMI Mortality Collaboration. Conventions as in Figure 1.
